# Systems and *in vitro* pharmacology profiling of diosgenin against breast cancer

**DOI:** 10.3389/fphar.2022.1052849

**Published:** 2023-01-04

**Authors:** Pukar Khanal, Vishal S. Patil, Vishwambhar V. Bhandare, Priyanka P. Patil, B. M. Patil, Prarambh S. R. Dwivedi, Kunal Bhattacharya, Darasaguppe R. Harish, Subarna Roy

**Affiliations:** ^1^ Department of Pharmacology, NGSM Institute of Pharmaceutical Sciences (NGSMIPS), Nitte (Deemed to be University), Mangalore, India; ^2^ ICMR-National Institute of Traditional Medicine, Belagavi, Karnataka, India; ^3^ Department of Microbiology, Shivaji University, Kolhapur, India; ^4^ Department of Pharmacology, KLE College of Pharmacy Belagavi, KLE Academy of Higher Education and Research (KAHER), Belagavi, India; ^5^ PRES’s Pravara Rural College of Pharmacy Pravaranagar, Loni, Maharashtra, India; ^6^ Pratiksha Institute of Pharmaceutical Sciences, Guwahati, Assam, India; ^7^ Royal School of Pharmacy, The Assam Royal Global University, Guwahati, Assam, India

**Keywords:** breast cancer, computational pharmacology, diosgenin, gene ontology analysis, system biology

## Abstract

**Aim:** The purpose of this study was to establish a mode of action for diosgenin against breast cancer employing a range of system biology tools and to corroborate its results with experimental facts.

**Methodology:** The diosgenin-regulated domains implicated in breast cancer were enriched in the Kyoto Encyclopedia of Genes and Genomes database to establish diosgenin-protein(s)-pathway(s) associations. Later, molecular docking and the lead complexes were considered for molecular dynamics simulations, MMPBSA, principal component, and dynamics cross-correlation matrix analysis using GROMACS v2021. Furthermore, survival analysis was carried out for the diosgenin-regulated proteins that were anticipated to be involved in breast cancer. For gene expression analyses, the top three targets with the highest binding affinity for diosgenin and tumor expression were examined. Furthermore, the effect of diosgenin on cell proliferation, cytotoxicity, and the partial Warburg effect was tested to validate the computational findings using functional outputs of the lead targets.

**Results:** The protein-protein interaction had 57 edges, an average node degree of 5.43, and a *p*-value of 3.83e-14. Furthermore, enrichment analysis showed 36 KEGG pathways, 12 cellular components, 27 molecular functions, and 307 biological processes. In network analysis, three hub proteins were notably modulated: *IGF1R*, *MDM2*, and *SRC*, diosgenin with the highest binding affinity with *IGF1R* (binding energy −8.6 kcal/mol). Furthermore, during the 150 ns molecular dynamics (MD) projection run, diosgenin exhibited robust intermolecular interactions and had the least free binding energy with *IGF1R* (−35.143 kcal/mol) compared to *MDM2* (−34.619 kcal/mol), and *SRC* (-17.944 kcal/mol). Diosgenin exhibited the highest cytotoxicity against MCF7 cell lines (IC_50_ 12.05 ± 1.33) µg/ml. Furthermore, in H_2_O_2_-induced oxidative stress, the inhibitory constant (IC_50_ 7.68 ± 0.51) µg/ml of diosgenin was lowest in MCF7 cell lines. However, the reversal of the Warburg effect by diosgenin seemed to be maximum in non-cancer Vero cell lines (EC_50_ 15.27 ± 0.95) µg/ml compared to the rest. Furthermore, diosgenin inhibited cell proliferation in SKBR3 cell lines more though.

**Conclusion:** The current study demonstrated that diosgenin impacts a series of signaling pathways, involved in the advancement of breast cancer, including FoxO, PI3K-Akt, p53, Ras, and MAPK signaling. Additionally, diosgenin established a persistent diosgenin-protein complex and had a significant binding affinity towards *IGF1R*, *MDM2*, and *SRC*. It is possible that this slowed down cell growth, countered the Warburg phenomenon, and showed the cytotoxicity towards breast cancer cells.

## Introduction

Breast cancer spreads through the inner layer of the milk gland or lobules and ducts (Sariego, 2010), and it is one of the second leading causes of mortality for women between the ages of 45 and 55 (Jemal et al., 2009). It includes age, iodine deficiency (Venturi, 2001; Aceves et al., 2005; Stoddard et al., 2008), high hormone levels (Russo and Russo, 2006; Yager and Davidson, 2006), and age-related (Steiner et al., 2008) risk factors. This incident potentially results in the breast being surgically removed entirely or in requiring chemo-, radio-, or hormone-therapy (HeraviKarimovi et al., 2006). However, these practises are preoccupied with multiple side effects that are not specific to the breast tumor. Furthermore, the currently used chemotherapeutic drugs in medical care result in anemia, exhaustion, mouth soreness, vomiting, and diarrhea. This suggests the requirement to discover a novel therapeutic agent against breast cancer.

Diosgenin is a phyto steroid sapogenin obtained from the hydrolysis by strong bases, acids, or enzymes of saponin. It is commercially used as a precursor to synthesis various hormones and steroid products like pregnenolone, and cortisone including progesterone (Marker and Krueger, 1940) for the early manufacture of combined oral contraceptive pills (Djerassi, 1992). Additionally, diosgenin has the potential to inhibit activated pro-inflammatory and pro-survival signaling pathways and promote the death of a variety of cancer cells (Shishodia and Aggarwal, 2006). Additionally, diosgenin inhibits the growth of oestrogen receptor-positive MCF-7 cells by activating caspase three and upregulating the p53 tumor suppressor gene. Further, BCL2 is downregulated in estrogen receptor-negative MDA-MB-231 breast cancer cells (Srinivasan et al., 2009). Although the potential of diosgenin to treat breast cancer has been demonstrated its interaction with proteins involved in the progenesis of breast cancer has yet to be investigated.

As a result, the current study focuses on locating the potential interactions of the diosgenin-regulated proteins and attributable pathways implicated in breast cancer using a range of system biology techniques *i.e.,* gene ontology (GO) analysis, molecular docking, and molecular dynamics (MD) simulations, and reinforcing its findings using diverse functional biomarkers using *in vitro* experiments in four distinct cell lines.

## Materials and methods

### Computational pharmacology

The detected diosgenin-regulated proteins were enriched to determine the altered pathways. Later, diosgenin was docked with proteins, and the proteins with the maximum diosgenin binding affinity were chosen for MD simulation. Furthermore, the diosgenin-regulated proteins involved in breast cancer were examined for survival and gene expression in normal and tumor cell lines, including the three hub proteins determined by molecular docking.

#### ADMET profile of diosgenin and its targets

**FIGURE 1:**
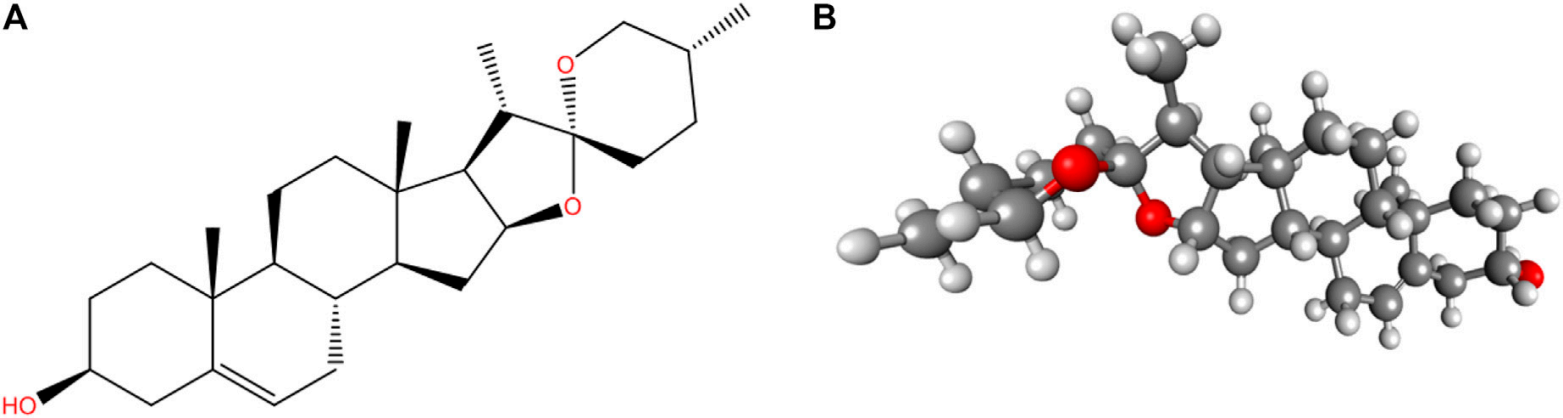
Structure of Diosgenin **(A)** 2D and **(B)** 3D (ball and stick). PubChem CID: 99474, Molecular Formula: C_27_H_42_O_3_, Synonyms: Nitogenin, UNII-K49P2K8WLX, (3β, 25R)-spirost-5-en-3-ol, Molecular Weight: 414.6.

Canonical SMILES of diosgenin (Figure 1) were retrieved from the PubChem database (https://pubchem.ncbi.nlm.nih.gov/) and ADME profile was predicted by SwissADME (Daina et al., 2017; http://www.swissadme.ch/) and ADMETlab 2.0 (Xiong et al., 2021; https://admetmesh.scbdd.com) and adverse effects from ADVERPred (Ivanov et al., 2018; http://www.way2drug.com/adverpred/).

Diosgenin-modulated targets were predicted using SwissTargetPrediction (Daina et al., 2019; http://www.swisstargetprediction.ch/), DIGEP-Pred (Lagunin et al., 2013; http://www.way2drug.com/ge/), and BindingDB (Liu et al., 2007; https://www.bindingdb.org/bind/index.jsp) and any duplicates were eliminated. In addition, the semantic type (neoplastic process) oestrogen receptor-positive breast cancer targets (UMLS CUI: C2938924), were retrieved from DisGeNET (https://www.disgenet.org/). Later, the diosgenin-modulated targets were compared with DisGeNET-recorded targets to trace the reported targets involved in oestrogen receptor-positive breast cancer using PivotTable (Microsoft excel 2007; https://www.microsoft.com/en-in/microsoft-365/excel).

#### GO and cluster analysis

The diosgenin-modulated targets involved in oestrogen receptor-positive breast cancer were queried in STRING (Szklarczyk et al., 2021; https://string-db.org/) ver 11.5 for “*Homo sapiens*” to trace three GO terms i.e., cellular components, molecular function, and biological processes. In addition, probable regulations of multiple pathways were also traced concerning the KEGG database (https://www.genome.jp/kegg/pathway.html) with whole genome statistical background. Also, the regulated proteins were concerned with subcellular location (COMPARTMENT), protein domains and features (InterPro), protein domain (Pfam), and tissue expression (TISSUES). Later, the protein-protein interaction (PPI) was also assessed for the cluster analysis via k means clustering to identify three lead distinct clusters.

### Network construction and analysis

The network between diosgenin, its targets (involved in oestrogen receptor-positive breast cancer), and the regulated pathways were constructed using Cytoscape ver 3.5.1 (Shannon et al., 2003; https://cytoscape.org/). The constructed network was recognized as directed and inspected by translating node size and color to low values corresponding to small sizes and low values corresponding to bright colors toward edge count. In addition, the edge size and color were mapped to edge betweenness, with low values corresponding to small sizes and low values equating to bright colors.

#### Molecular docking

The regulation of insulin-like growth factor 1 receptor (*IGF1R*), E3 ubiquitin-protein ligase Mdm2 (*MDM2*), and proto-oncogene tyrosine-protein kinase Src (*SRC*) were majorly triggered in the network interaction among diosgenin-target(s)-pathway(s). As a result, these three proteins were considered for molecular docking studies.

*Ligand preparation:* The ligand’s 3D conformation, diosgenin, was obtained from the PubChem database (https://pubchem.ncbi.nlm.nih.gov) and converted to .pdb using Discovery Studio Visualizer (https://discover.3ds.com/discover-studio-visualizer-download) *ver*. 2019. The ligand’s energy was minimized using the mmff94 force field (Halgren 1996) and saved in .pdbqt format.

*Preparation of macromolecule:* Diosgenin was docked against *IGF1R* (PDB: 3I81), *MDM2* (PDB: 3LBL), and *SRC* (PDB: 1O43); retrieved from a protein data bank maintained by the Research Collaboratory for Structural Bioinformatics (RCSB; https://www.rcsb.org). All proteins were refined and saved in .pdb format after eliminating all heteroatoms and pre-complex ligands.

*Ligand–protein docking:* Diosgenin was docked against the aforementioned targets using AutoDock Vina, which was run through the POAP pipeline (Trott and Olson 2010; Samdani and Vetrivel 2018; Patil et al., 2022). Nine different poses of ligand were obtained after docking. Docking results were analysed based on the binding affinity, and number of interactions as explained previously (Dwivedi et al., 2021; Badraoui et al., 2022). Further, the diosgenin pose with the lowest binding energy was chosen to visualize the ligand-protein interactions and perform MD simulation (Samdani and Vetrivel 2018)**.**

#### MD simulation

To examine the structural stabilities and intermolecular interactions of diosgenin with *IGF1R*, *MDM2*, and *SRC*, an all-atom MD simulation in an explicit solvent was performed for 150 ns. We used the GROMACS software package, *ver.* 2021.3 (https://www.gromacs.org/) and Amber *ff99SB-ildn* force field to run MD simulations (Berendsen et al., 2005; Saeed et al., 2020; Dwivedi et al., 2022). The topological parameters of the ligands and the entire complex were calculated using the AmberTool’s xleap module (https://ambermd.org/AmberTools.php) and the partial charges of the small molecules were calculated using an antechamber with a “bcc” charge model. The built systems were solvated in a rectangular box with 10.0 Å boundary conditions from the protein’s borders in all directions using the TIP3P water model. The required amounts of counter ions were introduced to the prepared systems to neutralize the charges. The steepest descent and conjugate gradient energy reduction methods were used to discover the least energy conformations of the near-global state. To equilibrate the systems for 1 ns, “Canonical (NVT) and isobaric (NPT) ensembles” were used. A modified Berendsen thermostat approach was utilized to keep the volume and temperature consistent during NVT equilibration (300 K). During NPT equilibration, a Parrinello-Rahman barostat was used to keep the pressure constant at 1 bar. Furthermore, the Particle Mesh Ewald approximation with a cut-off value of 1 nm was used to calculate the long-range electrostatic interactions, van der Waals, and Coulomb interactions. A similar LINear Constraint Solver method was used to constrain bond length. Every complex went through a 150 ns production run with coordinates recorded every 2 fs. Other software programs, in addition to the built-in gromacs tools, were utilized to perform specific analyses on the acquired trajectories as required.

#### Analysis of free binding energy of complexes utilizing molecular mechanics Poisson–Boltzmann surface area (MM-PBSA)

In MD simulations and thermodynamic calculations, the relative binding energy of a ligand-protein complex was employed to investigate the binding free energies. The relative binding free energy and its contribution to individual residues were calculated using the MM-PBSA method and the “g_mmpbsa” tool (Kumari et al., 2014; Dwivedi et al., 2022). The parameters for binding free energy calculations were taken from our previous study (Khanal et al., 2022). The binding free energy (ΔG) was calculated using 50 representative snapshots taken throughout the stable trajectory observed between 100 and 150 ns. The change in entropy (ΔS) was calculated using the Schlitter formula and finally, accurate binding free energy was calculated using the formula, ΔG = ΔH–TΔS, where, ΔG = Gibbs free energy, ΔH = enthalpy change, T = temperature (Kelvin), and ΔS = entropy change.

#### Principal component (PCA) and dynamic cross-correlation matrix (DCCM) analysis

PCA was performed over the stable MD trajectory to examine various forms of molecular motion (Amadei et al., 1993; Amadei et al., 1996; Bhandare and Ramaswamy 2018). To accomplish this, the “*least square fit*” to the reference structure was used to account for the molecular translational and rotational motion. The collection of eigenvectors obtained by diagonalizing a covariance matrix was produced by a linear transform of cartesian coordinate space to reflect the direction of the molecular motion. The energy contribution of each eigenvector to the motion was presented by the eigenvalue associated with the respective eigenvector. The “*time-dependent motions*” that the components carry out in an atomic vibrational mode were demonstrated by projecting the trajectory onto a particular eigenvector. The atomic vibrational components’ contribution to this form of coordinated motion was shown by the projection’s time average (Amadei et al., 1993; Van Aalten et al., 1995; Amadei et al., 1996; Bhandare and Ramaswamy 2018).

DCCM evaluates the magnitude of each pairwise cross-correlation coefficient to determine whether or not atomic pair motion is correlated *i.e.,* positive or negative (Khanal et al., 2021). We examined each DCCM component in this section, where C*ij* = 1 denotes the same period and phase (positive correlation), C*ij* = 0 indicates a lack of correlation, and C*ij* = −1 indicates a negative correlation between the fluctuations of *i* and j (Khanal et al., 2021).

#### Survival analysis of hub genes

To assess the prognostic values of hub genes of diosgenin-regulated oestrogen receptor-positive breast cancer targets, the Kaplan–Meier plotter breast cancer database (http://kmplot.com/analysis/) was used with *APP*, *AR*, *CDK4*, *CRHR1*, *CYP17A1*, *CYP19A1*, *CYP3A4*, *FASN*, *FGFR2*, *GRM1*, *IGF1R*, *LYN*, *MDM2*, *MDM4*, *NR3C1*, *PDGFRB*, *PRCP*, *PTPN1*, *RET*, *SRC*, and *STAT3* probe ID. Patients were divided into two groups with “*auto-detected best cutoff*”; overall survival was analyzed against *n* = 2,976 samples with a follow-up of 20 years of data. Also, the subtype analysis was restricted to lymph node status (*n* = 2,887), *ER* status (*n* = 2,789), *PGR* status (n = 2,662), *HER2* status (n = 2,875), *KI67* status (*n* = 1,360), Nottingham histologic grade (*n* = 2,917), and PAM50 subtype (n = 2,976) and cohort restriction *i.e.,* endocrine treated (*n* = 2,955) and chemo treated (*n* = 2,956). The difference among the groups was considered to be statistically significant if *p* < 0.05.

#### Gene expression analysis in tumor, normal and metastatic tissues

Herein, we evaluated the top three genes based on the log-rank test from survival analysis, and *in silico* molecular docking was evaluated for the gene expression in normal, tumor, and metastatic tissues using RNA sequence data in the platform of tumor, normal and metastatic samples. These data were analyzed using Kruskal Wallis and Dunn test.

### Experimental pharmacology

Through different *in vitro* pharmacology profiling, we investigated the influence of diosgenin on breast cancer cell lines. To begin, brine shrimp lethality (BSL) bioassay was used to assess its cytotoxicity and the effect was compared with doxorubicin. Later, the effect of diosgenin on breast cancer cell lines (MCF7, MDA-MB-231, SKBR3, and T47D) was compared to normal epithelial cell lines (Vero) by emphasizing on cell viability, proliferation, and Warburg effect.

#### BSL bioassay

The brine shrimp lethality bioassay was performed as explained by McLaughlin et al. (1998) with minor modifications. Here, *Artemia salina* Leach. eggs from Seamonk international Artemia cyst 003 were used for the assay. Briefly, 10–12 brine shrimps were incubated within the different concentrations of diosgenin and doxorubicin (prepared in seawater) for 24 h. Controls were used without the test agents. After 24 h, the survived shrimps were counted and the % cytotoxicity was calculated as

$$\%\;cytotoxicity=\left(\frac{Total\;shrimps\;added-live\;shrimps}{Total\;shrimps}\right)\times 100$$

The LC_50_ was calculated using a linear regression curve.

#### *In vitro* MTT assay tumor and non-tumor cell lines

The cytotoxic activity of diosgenin and doxorubicin on tumor and normal cell lines was performed using an MTT assay (Mosmann, 1983) with minor modifications. Briefly, cell lines were plated onto 96-well flat-bottom plates, maintaining the cell density (20,000 cells/well), and were allowed to proliferate (24 h). After that, the cells were treated with different concentrations of diosgenin and doxorubicin maintaining the final volume of 250 µL after adding DMEM media (supplemented with 3% FBS) and incubated (37°C, 48 h, 5% CO_2_). Next, 20 μL of MTT reagent was added and incubated (37°C in 5% CO_2_, 4 h). After incubation, the wells were washed (PBS, 3X) to discard the MTT. Then, formazan crystals were dissolved in DMSO (99.5% v/v, 100 μL) by gentle shaking. The absorbance was then recorded (570 nm) using an ELISA plate reader. The cell viability was calculated as

$$\%\;viability=\left(\frac{Absorbance\;of\;control-Absorbance\;of\;sample}{Absorbance\;of\;control}\right)\times 100$$

#### *In vitro* scratch assay

*In vitro* scratch assay was performed as explained by Bolla et al (2019) with minor modifications. Briefly, a stock solution (200 μg/ml) of diosgenin and doxorubicin was prepared and sterilized by filtering using a sterile membrane filter (0.22 µm). Later the solution was diluted using geometric series up to 1.56 μg/ml. This series of concentrations were chosen based on the number of experiments (trial and error). All the cells were seeded (2 × 10^5^ cells/well) in 12-well tissue culture plates to obtain the confluence of 80–90% after 24 h of culture. After 24 h of post-seeding, the cell monolayers were scraped to create scratches of (300 µm). The detached cells and debris were washed with phosphate buffer. The media containing the samples were added to each well. Suitable controls were used by adding the minimal media and the scratch coverage was recorded at 0, 12, 24, 48, and 72 h after sample addition. The percentage scratch coverage was calculated using the following formula

$$\%\;scratch\;coverage=\left(\frac{Scratch\;length\;at\;\left(0\;\min-t\;\min\right)}{Scratch\;length\;at\;0\;\min}\right)\times 100$$

#### Effect of diosgenin on Warburg effect

The effect of diosgenin on the Warburg phenomena was evaluated by evaluating glucose uptake *via* the above-mentioned cancer cell lines vs*.* normal cell lines (Vero). Initially, the cells were grown in six well plates and incubated (37°C, 48 h) in a CO_2_ incubator. After the formation of the confluent monolayer, the culture was renewed (DMEM consisting of 0.2% BSA) and again incubated (37°C, 18 h) in the CO_2_ incubator. After incubation, the media was discarded and washed with KRP buffer. The cells were then treated with diosgenin and metformin in the presence of insulin followed by the addition of glucose (1 M) and incubated (30 min). The remaining amount of glucose was quantified from the supernatant. The percentage glucose uptake was calculated as the difference between the initial and final glucose content in the incubated medium (Gupta et al., 2009).

### Statistical analysis

For the enrichment analysis, the whole-genome statistical background was used. The inhibitory constant (IC_50_) and effective concentration (EC_50_) were calculated using linear regression in GraphPad Prism (https://www.graphpad.com/) *ver* 5.0.

## Results

### Computational pharmacology

#### Diosgenin ADMET profile and its targets

Diosgenin has molecular weight of 414.62 g/mol with 3 *H*-bond acceptors and 1 *H*-bond donor. Diosgenin was predicted to possess the high human intestinal absorption (>30). In addition, it also showed the plasma protein binding, fraction unbound in plasma, and its volume of distribution 97.743%, 1.872%, and 1.695 L/kg, respectively. It was predicted as no inhibitory action towards *CYP1A2*, *CYP2C19*, *CYP2C9*, *CYP2D6*, and *CYP3A4* and showed high clearance rate of 23.332 ml/min/kg. In addition, it was not predicted for any side effects based on the ADVERPred server projection.

Diosgenin was predicted to regulate 105 targets in SwissTargetPrediction and four in DIGEP-Pred and BindingDB. The predicted targets in BindingDB were in common with SwissTargetPrediction. Similarly, a total of 510 different targets were recorded in DisGeNET for the neoplastic process of oestrogen receptor-positive breast cancer targets (UMLS CUI: *C2938924*). In general, the Diosgenin targeted 21 different proteins involved in breast cancer compared to recorded targets in DisGeNET (UMLS CUI: *C2938924*); Supplementary Figure S1.

#### Gene set enrichment analysis

**FIGURE 2:**
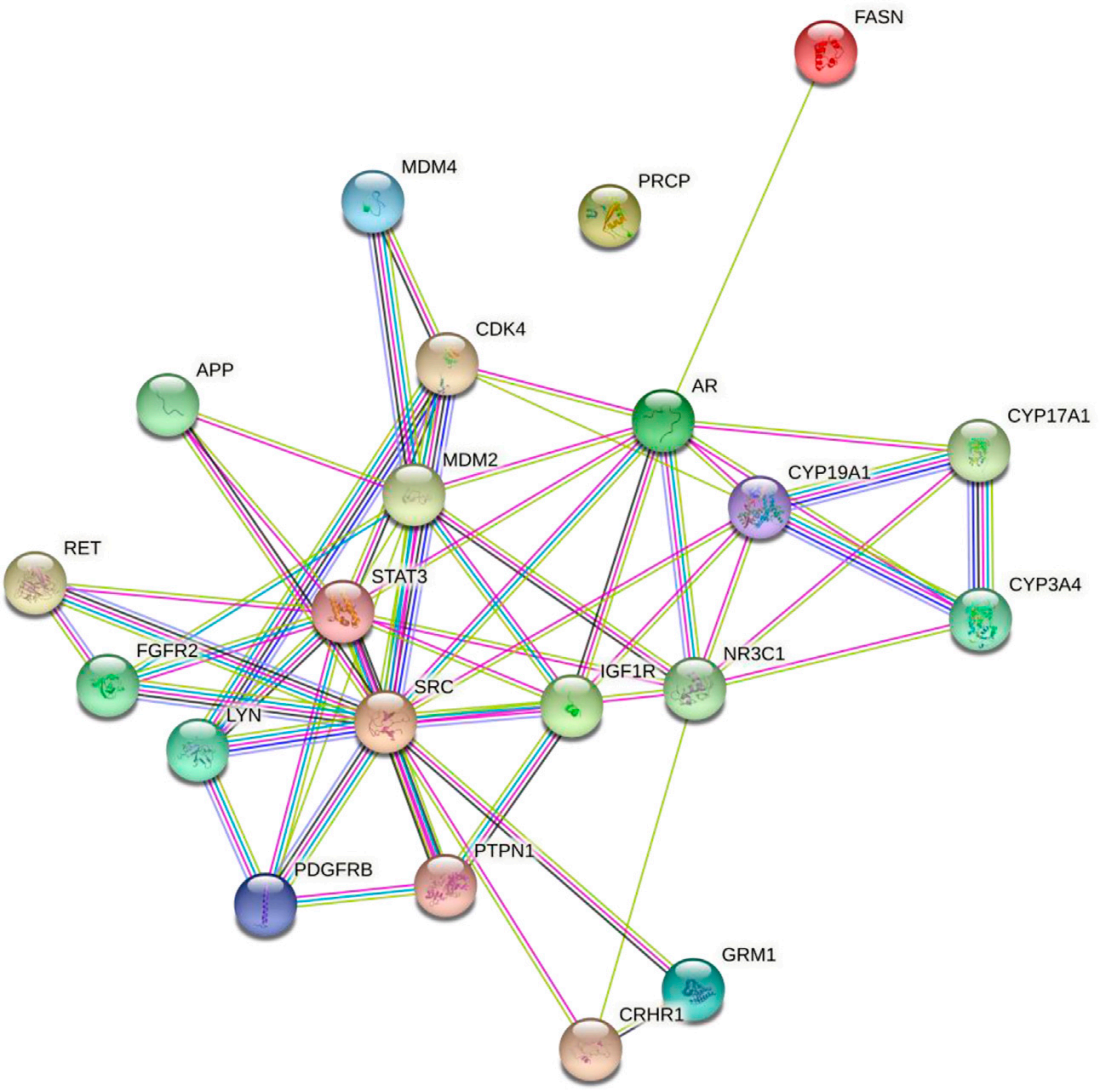
Protein-protein interaction of the diosgenin-triggered protein. Node color; 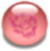
colored nodes: query proteins and first shell of interactors, 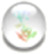
white nodes: second shell of interactors, Node content; 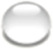
empty nodes: proteins of unknown 3D structure, 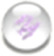
filled nodes: some 3D structure is known or predicted, Known Interactions; 
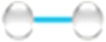
 *from curated databases,* 
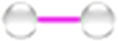
 *experimentally determined*, Predicted Interactions; 
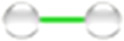
 *gene neighborhood,* 
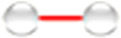
 *gene fusions,* 
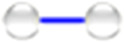
 *gene co-occurrence* and Others; 
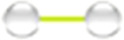
 *text mining*, 
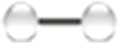
 *co-expression,* 
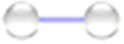
 *protein homology.*

The interaction of diosgenin-targeted 21 proteins had a total of 57 edges, 5.43 average node degree, 0.701 average local clustering coefficient, 17 edges (expected), and 3.83e-14 enrichment *p*-value (Figure 2).

#### KEGG enrichment analysis

**FIGURE 3:**
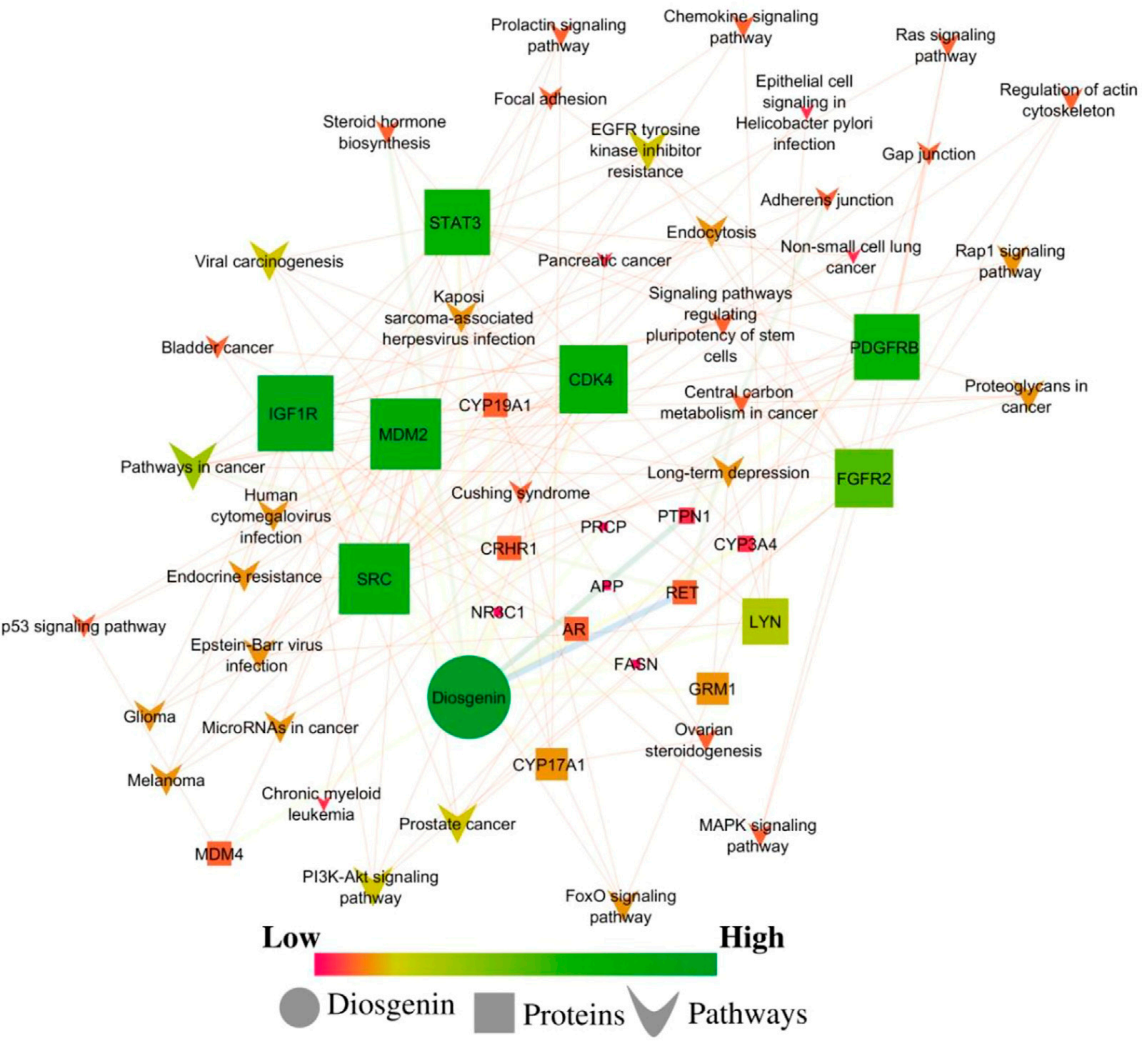
Network interaction of diosgenin-regulated proteins and respective pathways. Pink color presents a low edge count and green color presents a high edge count. A large node size presents a higher edge count and a smaller edge count presents a lower edge count.

**FIGURE 4:**
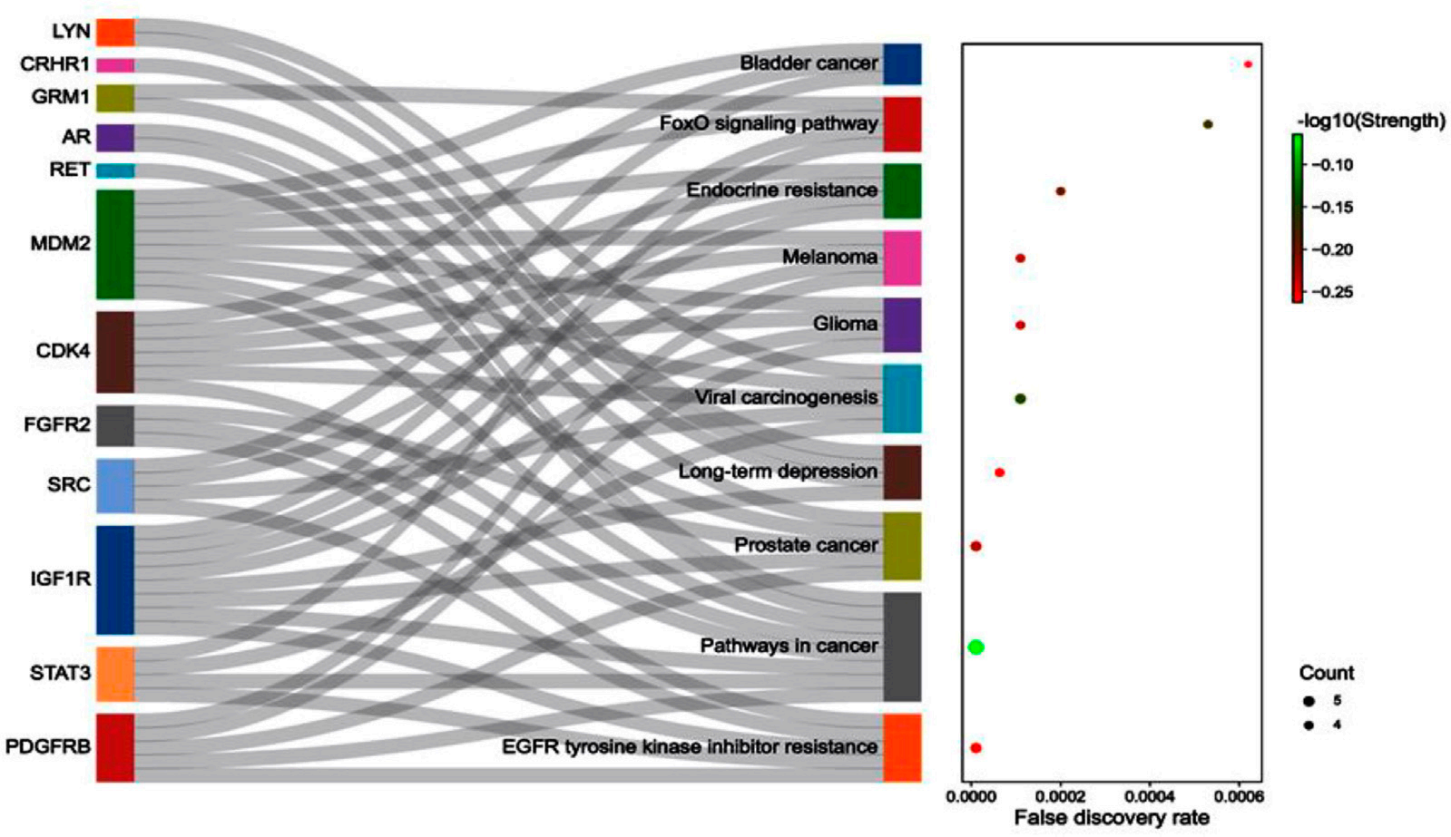
The associated protein-pathways interaction with respective false discovery rate and strength.

Concerning the KEGG record, the PPI reflected the regulation of 36 different pathways in which *EGFR* tyrosine kinase inhibitor resistance (*hsa01521*) had the lowest false discovery rate *i.e.,* 1.06E-05 *via* the regulation of five genes *i.e., PDGFRB*, *STAT3*, *IGF1R*, *SRC*, and *FGFR2* against 78 background genes at 1.78 strength. Additionally, eight different pathways were detected for keyword “*cancer*” *i.e.* pathways in cancer; *hsa05200* (regulated eight genes *i.e. CDK4*, *MDM2*, *PDGFRB*, *STAT3*, *IGF1R*, *RET*, *AR*, and *FGFR2* against 517 background genes at 1.16 strength and 1.06E-05 false discovery rate), prostate cancer; *hsa05215* (regulated five genes *i.e. MDM2*, *PDGFRB*, *IGF1R*, *AR*, and *FGFR2* against 96 background genes at 1.69 strength and 1.06E-05 false discovery rate), bladder cancer; *hsa05219* (regulated three genes *i.e. CDK4*, *MDM2*, and *SRC* against 41 background genes at 1.83 strength and 0.00062 false discovery rate), microRNAs in cancer; *hsa05206* (regulated four genes *i.e. MDM2*, *PDGFRB*, *STAT3*, and *MDM4* against 160 background genes at 1.37 strength and 0.00099 false discovery rate), proteoglycans in cancer; *hsa05205* (regulated four genes *i.e. MDM2*, *STAT3*, *IGF1R*, and *SRC* against 196 background genes at 1.28 strength and 0.0015 false discovery rate), central carbon metabolism in cancer; *hsa05230* (regulated three genes *i.e. PDGFRB*, *RET*, and *FGFR2* against 69 background genes at 1.61 strength and 0.0015 false discovery rate), non-small cell lung cancer; *hsa05223* (regulated two genes *i.e. CDK4* and *STAT3* against 68 background genes at 1.44 strength and 0.0349 false discovery rate), and pancreatic cancer; *hsa05212* (regulated two genes *i.e. CDK4* and *STAT3* against 73 background genes at 1.41 strength and 0.0387 false discovery rate). Also, a total of 129 genes were modulated in 36 different pathways in which insulin-like growth factor-1 receptor (*IGF1R*) was majorly triggered in 18 different pathways *i.e., EGFR* tyrosine kinase inhibitor resistance; *hsa01521*, pathways in cancer; *hsa05200*, prostate cancer; *hsa05215*, long-term depression; *hsa04730*, glioma; *hsa05214*, melanoma; *hsa05218*, endocrine resistance; *hsa01522*, ovarian steroidogenesis; *hsa04913*, signaling pathways regulating pluripotency of stem cells; hsa04550, adherens junction; *hsa04520*, proteoglycans in cancer; *hsa05205*, endocytosis; *hsa04144*, focal adhesion; *hsa04510*, and Ras; *hsa04014*, MAPK; *hsa04010*, FoxO; *hsa04068*, PI3K-Akt; *hsa04151*, and Rap1 signaling pathway; *hsa04015* (Supplementary File S1; Supplementary Sheet S1). The modulated genes in the KEGG pathways were observed to be common with three GO terms *i.e.,* cellular components, molecular function, and biological processes (Supplementary Figure S2); detailed below. Further, the interaction between the diosgenin-modulated targets and triggered pathways is presented in Figure 3. The associated protein-pathways interaction with respective false discovery rate and strengths is presented in Figure 4.

#### GO analysis

**FIGURE 5:**
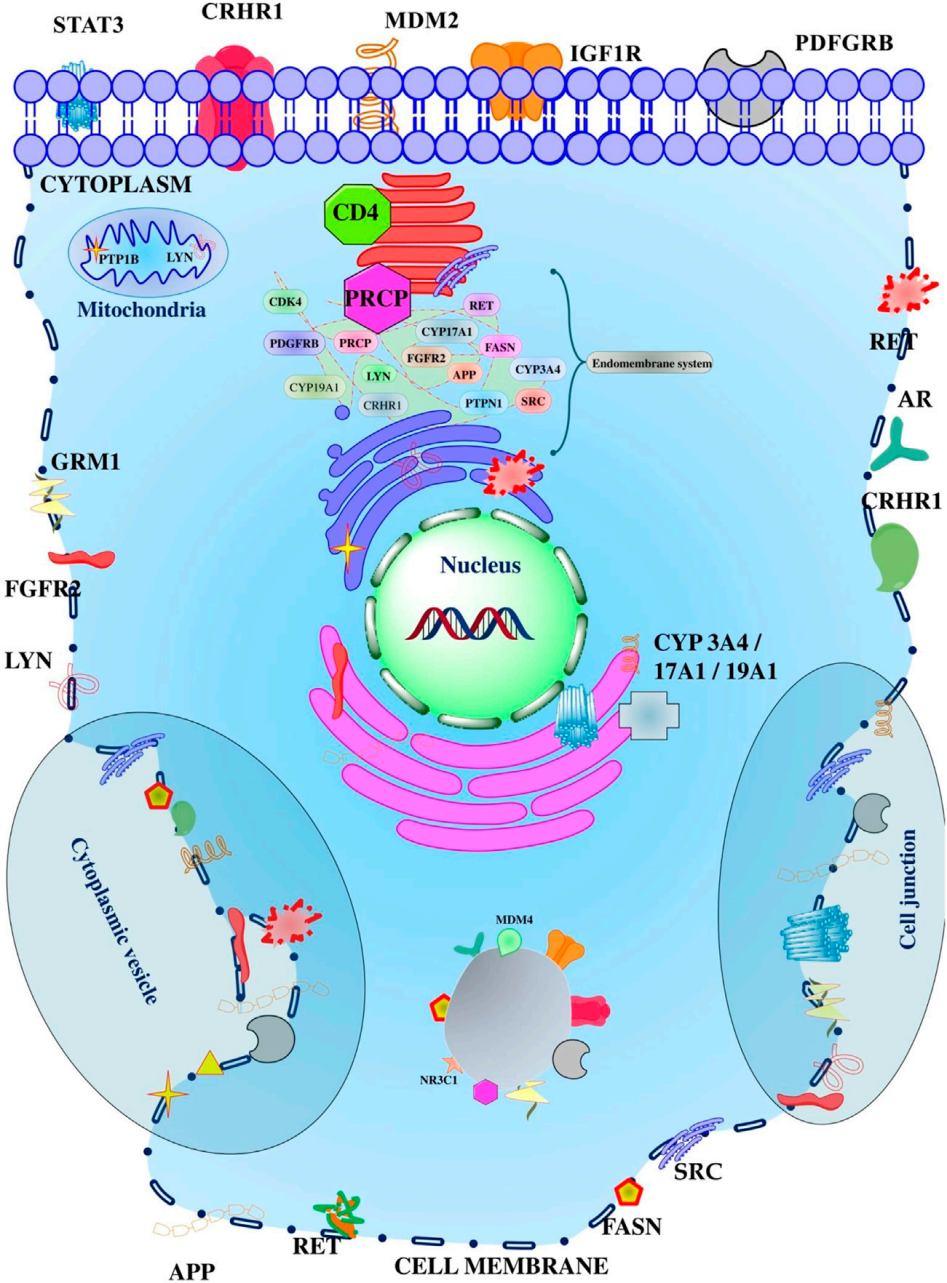
Different proteins affected by the diosgenin in the various compartment of the cell. The cellular organelles presented reflect the membrane-bounded organelle or intracellular organelle.

GO analysis identified 12 GO terms for cellular components in which receptor complex; GO:*0043235* had the lowest false discovery rate *i.e.,* 0.00025 regulated seven genes *i.e., PDGFRB*, *IGF1R*, *APP*, *RET*, *GRM1*, *FGFR2*, and *LYN* against 381 genes at 1.23 strength. Also, a total of 21 genes were triggered in multiple cellular components in which tyrosine-protein kinase Lyn (*LYN*) was majorly triggered in 11 cellular components except for cytoplasmic vesicles; GO:*0031410* (Supplementary File S1; Supplementary Sheet S1). In addition, the Pearson *p*-value for cellular components was 0.795 strength vs*.* false discovery rate, 0.0003 strength vs*.* observed gene count, 0.8093 false discovery rate vs*.* observed gene count, 2.684e-004 observed gene count vs*.* strength, and 0.809 observed gene count vs*.* false discovery rate. In addition, the minimum observed Pearson r was −0.084 and the maximum was 1.000 (Supplementary Figure S3). The proteins modulated by the diosgenin in the different cellular compartments are presented in Figure 5.

Likewise, 27 different molecular functions GO terms were identified *via* the PPI in which protein tyrosine kinase activity; GO:*0004713* had the lowest false discovery rate *i.e.,* 3.53E-05 to trigger six genes *i.e., PDGFRB*, *IGF1R*, *RET*, *SRC*, *FGFR2*, and *LYN* against 137 background genes at 1.61 strength. Herein, a total of 22 genes were triggered in 27 molecular functions in which proto-oncogene tyrosine-protein kinase Src (*SRC*) was involved in 18 molecular functions except hormone binding; GO:*0042562*, transmembrane receptor protein tyrosine kinase activity; GO:*0004714*, transition metal ion binding; GO:*0046914*, steroid hydroxylase activity; GO:*0008395*, oxygen binding; GO:*0019825*, nuclear receptor activity; GO:*0004879*, peptide hormone binding; GO:*0017046*, platelet-derived growth factor receptor binding; GO:*0005161*, and steroid-binding; GO:*0005496* (Supplementary File S1; Supplementary Sheet S3). Herein, the correlation *p* values were 0.130 strength vs*.* false discovery rate, 2.0510e-009 strength vs*.* observed gene count, and 0.753 false discovery rate vs*.* observed gene count. Within the interaction of strength, false discovery rate, and observed gene count, −0.877 and 1.000 were minimum and maximum r values for molecular function (Supplementary Figure S3).

Similarly, a total of 307 biological processes was traced due to PPI in which cellular response to oxygen-containing compound; GO:*1901701* had the lowest false discovery rate *i.e.,* 2.72E-09 in modulating 14 genes *i.e., NR3C1*, *CDK4*, *MDM2*, *PDGFRB*, *STAT3*, *IGF1R*, *APP*, *RET*, *PTPN1*, *SRC*, *AR*, *CRHR1*, *FGFR2*, and *LYN* against 1,055 background proteins at 1.09 strength. In addition, a total of 21 genes were triggered for 307 biological processes *via* the 21 proteins associated with breast cancer in which *SRC* was involved and linked with 213 biological processes. In addition, the order of triggered genes in multiple pathways was as *CYP17A1* (**17**) < *CYP3A4* (**22**) < *PRCP* (**33**) < *CRHR1* (**35**) < *FASN* (**42**) < *GRM1* (**52**) < *CYP19A1* (**59**) < *NR3C1* (**60**) < *MDM4* (**71**) < *CDK4* (**111**) < *IGF1R* (**113**) < *PTPN1* (**114**) < RET (**130**) < *MDM2* (**134**) < *AR* (**139**) < *STAT3* (**140**) < *FGFR2* (**149**) < *PDGFRB* (**160**) < *LYN* (161) < *APP* (**198**) < *SRC* (**213**). The detailed biological processes of participation of the above genes are supplemented in Supplementary File S1; Supplementary Sheet S4. The Pearson correlation *p*-value was found to be 0.034 strength vs*.* false discovery rate, 1.0622e-063 strength vs*.* observed gene count, and 8.1767e-017 false discovery rate vs*.* observed gene count. In addition, the minimum Pearson *r* values for biological processes were -0.779 and 1.000 respectively (Supplementary Figure S3).

#### Compartments enrichment analysis

The PPI reflected the modulation of proteins in 16 different compartments in which the intrinsic component of plasma membrane; GOCC:*0031226* had the lowest false discovery rate *i.e.,* 0.00027 in regulating nine genes *i.e., PDGFRB*, *STAT3*, *IGF1R*, *APP*, *RET*, *GRM1*, *CRHR1*, *FGFR2*, and *LYN* against 841 background genes at 1 strength. Herein, a total 21 were triggered in 16 compartments in which *LYN* was traced in all; the order of genes regulation in multiple enriched compartments was *MDM4* (**5**) < *NR3C1* (**6**) = *AR* (**6**) < *CYP17A1* (**7**) < *CYP19A1* (**8**) = *CYP3A4* (**8**) = *CDK4* (**8**) = *GRM1* (**8**) = *MDM2* (**8**) < *FASN* (**9**) = *PDGFRB* (**9**) = *PRCP* (**9**) = *PTPN1* (**9**) = *SRC* (**9**) < *CRHR1* (**12**) = *FGFR2* (**12**) < *IGF1R* (**13**) < *RET* (**14**) = *STAT3* (**14**) < *APP* (**15**) < *LYN* (**16**); Supplementary File S1; Supplementary Sheet S5.

#### InterPro enrichment analysis

Protein-protein enrichment analysis traced seven InterPro in which tyrosine-protein kinase, catalytic domain; *IPR020635* was traced with minimum false discovery rate *i.e.,* 8.42E-06 to regulate six genes *i.e., PDGFRB*, *IGF1R*, *RET*, *SRC*, *FGFR2*, and *LYN* against 79 background genes at 1.85 strength. Herein, in the enrichment of InterPro analysis, a total of nine genes were triggered in order as *MDM2* (**1**) = *MDM4* (**1**) < *CDK4* (**3**) < *FGFR2* (**6**) = *IGF1R* (**6**) = *LYN* (**6**) = *PDGFRB* (**6**) = *RET* (**6**) = *SRC* (**6**); Supplementary File S1; Supplementary Sheet S6.

#### Pfam enrichment analysis

Enrichment analysis concerning to Pfam database traced the regulation of protein kinase domain; *PF00069* and protein tyrosine kinase; *PF07714* at the false discovery rate of 0.0068 to regulate seven genes *i.e., CDK4*, *PDGFRB*, *IGF1R*, *RET*, *SRC*, *FGFR2*, and *LYN* at 1.13 strength against 486 and 481 background genes respectively; Supplementary File S1; Supplementary Sheet S7.

#### Tissues enrichment analysis

Enrichment analysis of PPI for TISSUES traced 13 different TISSUES terms in which erythroleukemia cell; BTO:*0000426* was traced with the lowest false discovery rate *i.e.,* 0.0088 in regulating five genes *i.e., MDM2*, *STAT3*, *PTPN1*, *SRC*, and *LYN* at 1.32 strength against 225 background genes. Herein, there was a regulation of 21 genes *i.e. APP*, *AR*, *CDK4*, *CRHR1*, *CYP17A1*, *CYP19A1*, *CYP3A4*, *FASN*, *FGFR2*, *GRM1*, *IGF1R*, *LYN*, *MDM2*, *MDM4*, *NR3C1*, *PDGFRB*, *PRCP*, *PTPN1*, *RET*, *SRC*, and *STAT3* in which nuclear receptor subfamily 3, group C, member 1 (*NR3C1*) was majorly triggered in 10 different TISSUES *i.e.* bone marrow cancer cell (BTO:*0000583*), organism form (BTO:*0000284*), leukemia cell (BTO:*0001271*), fibroblast (BTO:*0000452*), liver (BTO:*0000759*), whole body (BTO:*0001489*), reproductive system (BTO:*0000081*), embryonic structure (BTO:*0000174*), endocrine gland (BTO:*0001488*) and connective tissue (BTO:*0000421*); Supplementary File S1; Supplementary Sheet S8.

#### Cluster analysis

K means analysis of PPI against the whole genome of *Homo sapiens* traced three sets of clusters which are indicated by red (cluster 1; included *AR*, *CYP17A1*, *CYP19A1*, *CYP3A4*, *FASN*, *NR3C1*, and *PRCP*), green (cluster 2; included *APP*, *CDK4*, *CRHR1*, *FGFR2*, *GRM1*, *IGF1R*, *LYN*, *PDGFRB*, *PTPN1*, *RET*, *SRC*, and *STAT3*) and blue (cluster 3; included *MDM2* and *MDM4*); Supplementary Table S1.

In cluster 1, the interaction of seven nodes corresponded to 11 edges with 3.14 average node degree, 0.8 average local clustering coefficients, 1 expected edge, and 1.07e-09 PPI enrichment *p*-value. Herein, a total of nine molecular functions were traced in which steroid hydroxylase activity (GO:*0008395*) and oxygen binding (GO:*0019825*) scored the lowest false discovery rate *i.e.,* 0.0013 *via* the regulation of three genes *i.e., CYP3A4*, *CYP17A1*, and *CYP19A1* against 36 background genes at 2.37 strength. Similarly, Steroid binding (GO:*0005496*) showed 0.0082 false discovery rate and regulated three genes *i.e., NR3C1*, *CYP3A4*, and *AR* against 104 background genes at a strength of 1.91. Similarly, two KEGG pathways *i.e.,* steroid hormone biosynthesis (*hsa00140*) and ovarian steroidogenesis (*hsa04913*) were traced. Herein, steroid hormone biosynthesis was identified with a false discovery rate of 0.00048 to regulate three genes *i.e., CYP3A4*, *CYP17A1*, and *CYP19A1* against 59 background genes at 2.15 strength. In addition, ovarian steroidogenesis regulated two genes *i.e., CYP17A1* and *CYP19A1* against 50 genes at 2.05 strength and 0.0327 false discovery rate. Also, two different biological processes *i.e.,* androgen metabolic process (GO:*0008209*) and organic cyclic compound biosynthetic process (GO:*1901362*) were identified to regulate three genes *i.e., CYP3A4*, *CYP17A1*, and *CYP19A1* against 27 background genes at 2.47 strength and 0.002 false discovery rate and six genes (*NR3C1*, *FASN*, *CYP3A4*, *CYP17A1*, *AR*, and *CYP19A1I*) against 1,211 background genes at 1.14 strength and 0.0033 respectively (Supplementary File S2; Supplementary Sheet S1).

In cluster 2, 12 protein interactions traced 25 edges with 4.17 average node degree, 0.843 average local clustering coefficient, seven expected edges, and 3.83e-07 PPI enrichment *p*-value. Herein, a total of 25 enriched cellular components were traced in which receptor complex (GO:*0043235*), an integral component of the plasma membrane (GO:*0005887*), and cell junction (GO:*0030054*) were majorly enriched at a false discovery rate of 2.03E-06, 0.001, and 0.0033 and strength of 1.48, 0.91, and 0.8 respectively. Herein, the receptor complex was enriched with seven genes *i.e., PDGFRB*, *IGF1R*, *APP*, *RET*, *GRM1*, *FGFR2*, and *LYN*, an integral component of the plasma membrane with eight genes *i.e., PDGFRB*, *IGF1R*, *APP*, *RET*, *GRM1*, *CRHR1*, *FGFR2*, and *LYN* and cell junction with eight genes *i.e., CDK4*, *PDGFRB*, *STAT3*, *APP*, *GRM1*, *SRC*, *FGFR2*, and *LYN*. Also, a total of 21 enriched molecular functions were traced for cluster 2 in which protein tyrosine kinase activity; GO:*0004713* (regulated six genes *i.e. PDGFRB*, *IGF1R*, *RET*, *SRC*, *FGFR2*, and *LYN* against 137 background genes at 1.85 strength and 6.36E-07 false discovery rate), insulin receptor binding; GO:*0005158* (regulated four genes *i.e. IGF1R*, *APP*, *PTPN1*, and *SRC* against 24 background genes at 2.43 strength and 4.29E-06 false discovery rate) and ephrin receptor binding; GO:*0046875* (regulated four genes *i.e. APP*, *PTPN1*, *SRC*, and *LYN* against 28 background genes at 2.37 strength and 5.02E-06 false discovery rate). A total of 45 KEGG pathways were traced within the PPI of cluster 2 in which *EGFR* tyrosine kinase inhibitor resistance; *hsa01521* (regulated five genes *i.e. PDGFRB*, *STAT3*, *IGF1R*, *SRC*, and *FGFR2* against 78 background genes at 2.02 strength and 4.26E-07 false discovery rate), long-term depression; *hsa04730* (regulated four genes *i.e. IGF1R*, *GRM1*, *CRHR1*, and *LYN* against 59 background genes at 2.04 strength and 1.07E-05 false discovery rate), and pathways in cancer; *hsa05200* (regulated six genes *i.e. CDK4*, *PDGFRB*, *STAT3*, *IGF1R*, *RET*, and *FGFR2* against 517 background genes at 1.28 strength and 4.31E-05 false discovery rate) were identified to be top three majorly triggered pathways. Similarly, a total of 222 biological processes were traced within the PPI of cluster 2 in which cellular response to oxygen-containing compound; GO:*1901701* (regulated 11 genes *i.e. CDK4*, *PDGFRB*, *STAT3*, *IGF1R*, *APP*, *RET*, *PTPN1*, *SRC*, *CRHR1*, *FGFR2*, and *LYN* against 1,055 background genes at 1.23 strength and 2.37E-09 false discovery rate), regulation of protein serine/threonine kinase activity; GO:*0071900* (regulated nine genes *i.e. CDK4*, *PDGFRB*, *IGF1R*, *APP*, *RET*, *GRM1*, *PTPN1*, *SRC*, and *LYN* against 521 genes at 1.45 strength and 1.31E-08 false discovery rate), and regulation of protein phosphorylation; GO:*0001932* (regulated 11 genes *i.e. CDK4*, *PDGFRB*, *STAT3*, *IGF1R*, *APP*, *RET*, *GRM1*, *PTPN1*, *SRC*, *FGFR2*, and *LYN* against 1,459 background genes at 1.09 strength and 1.93E-08 false discovery rate (Supplementary File S2; Supplementary Sheet S2).

In cluster 3 the interaction of the nodes had 1 edge count, average node degree, and average local clustering coefficient with 0.0398 PPI enrichment *p*-values. Here, only two KEGG pathways and eight biological processes were traced. In KEGG pathways, the p53 signaling pathway; *hsa04115,* and microRNAs in cancer; *hsa05206* were associated with two genes *i.e., MDM2* and *MDM4* against 72 and 160 background genes, 2.43 and 2.09 strength and 0.0064 and 0.0155 false discovery rate respectively. Further, interaction between *MDM2* and *MDM4* triggered eight biological processes *i.e.* atrioventricular valve morphogenesis; GO:*0003181* (24 background genes at 2.91 strength and 0.0274 false discovery rate), endocardial cushion morphogenesis; GO:*0003203* (34 background genes at 2.76 strength and 0.0274 false discovery rate), ventricular septum development; GO:*0003281* (73 background genes at 2.43 strength and 0.0274 false discovery rate), atrial septum development; GO:*0003283* (23 background genes at 2.93 strength and 0.0274 false discovery rate), DNA damage response, signal transduction by p53 class mediator resulting in cell cycle arrest; GO:*0006977* (59 background genes, 2.52 strength and 0.0274 false discovery rate), negative regulation of cell cycle arrest; GO:*0071157* (23 background genes, 2.93 strength and 0.0274 false discovery rate), regulation of signal transduction by p53 class mediator; GO:*1901796* (182 background genes, 2.03 strength, and 0.0376 false discovery rate), and cellular response to hypoxia; GO:*0071456* (189 background genes, 2.02 strength and 0.0395 false discovery rate); Supplementary File S2; Supplementary Sheet S3.

#### Diosgenin-targets-protein network analysis

The combined interaction between the diosgenin, its targets, and regulated pathways traced *IGF1R*, *MDM2*, *SRC*, *CDK4*, and *PDGFRB* as the top five lead hub proteins. In addition, pathways in cancer; *hsa05200*, *EGFR* tyrosine kinase inhibitor resistance; *hsa01521* prostate cancer; *hsa05215*, viral carcinogenesis; *hsa05203*, and PI3K-Akt signaling pathway; *hsa04151* were traced as the top five lead hub pathways modulated within diosgenin-targets-pathways interactions.

In diosgenin-targets-pathways interactions, none of the nodes were single or undirected. Herein, two pathways’ nodes *i.e.,* adherens junction and central carbon metabolism in cancer had a maximum average shortest path length *i.e.,* 2.67. Herein, 33 nodes *i.e.* diosgenin, PI3K-Akt signaling pathway, *EGFR* tyrosine kinase inhibitor resistance, viral carcinogenesis, prostate cancer, endocrine resistance, proteoglycans in cancer, glioma, melanoma, human cytomegalovirus infection, endocytosis, Rap1 signaling pathway, FoxO signaling pathway, Kaposi sarcoma-associated herpesvirus infection, Epstein-Barr virus infection, MicroRNAs in cancer, long-term depression, focal adhesion, bladder cancer, signaling pathways regulating pluripotency of stem cells, Ras signaling pathway, *MAPK* signaling pathway, regulation of actin cytoskeleton, chemokine signaling pathway, p53 signaling pathway, prolactin signaling pathway, gap junction, ovarian steroidogenesis, Cushing syndrome, chronic myeloid leukemia, non-small cell lung cancer, pancreatic cancer, and epithelial cell signaling in *Helicobacter pylori* infection had the maximum closeness centralities *i.e.* 1. Further, the *CYP3A4* node had the maximum clustering coefficient *i.e.,* 0.5. Likewise, three pathways nodes *i.e.,* adherens junction, central carbon metabolism in cancer, and pathways in cancer had maximum eccentricity *i.e.,* 3. In addition, node diosgenin had the maximum stress *i.e.,* 101 followed by 21°, 0.03 betweenness centrality, 21 directed nodes, 0.85 radiality, 22 edge count, and, 19 outdegrees. Further, four nodes i.e. *NR3C1*, *PRCP*, *APP*, and *FASN* had maximum neighborhood connectivity *i.e.,* 22. In addition, the *AR* node had the maximum topological coefficient *i.e.,* 0.79 and *IGF1R* had maximum indegree *i.e.,* 19 (Supplementary File S3; Supplementary Sheet S1).

**FIGURE 6:**
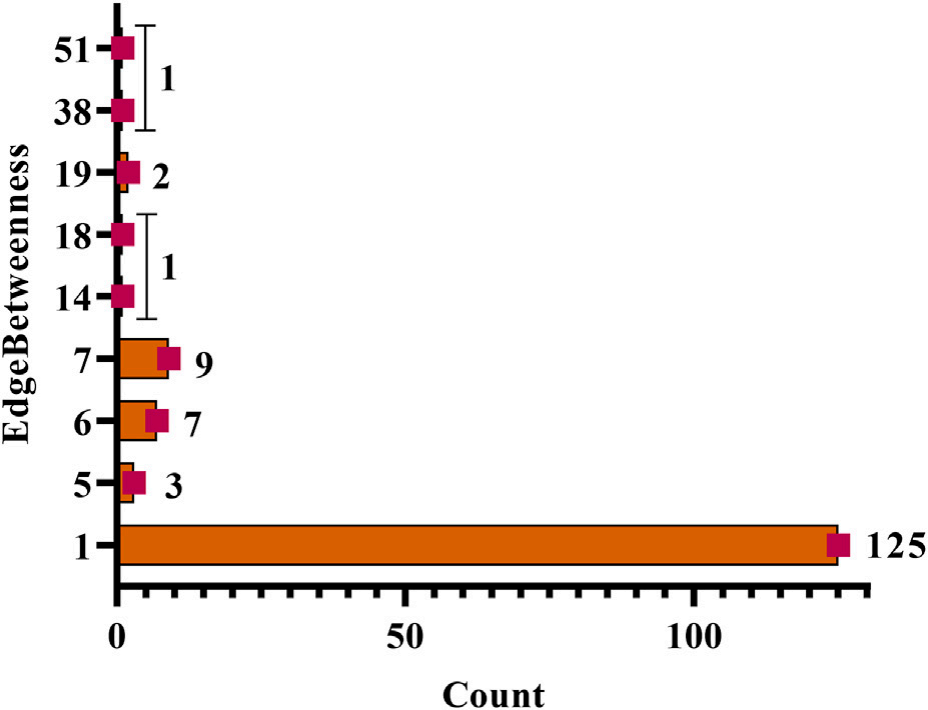
Edgebetweenness of network interaction and node interaction count. The highest edge betweenness was 51 (1 interaction) and the lowest was 1 (125 interactions).

In a network a total of nine categories of edge betweenness were traced *i.e.,* 1 (125 interactions), 5 (3 interactions), 6 (7 interactions), 7 (9 interactions), 19 (2 interactions), 14, 18, 38, and 51 had the 1 interaction (Figure 6). Herein, within the whole network, 51 edge betweenness was within the *RET* and diosgenin interaction, 38 with *PTP1B* and diosgenin interaction, 19 with adherens junction interaction with *PTPN1*, and central carbon metabolism in cancer interaction with *RET*, 18 with steroid hormone biosynthesis with diosgenin, and 14 with pathways in cancer with *RET* (Supplementary File S3; Supplementary Sheet S2).

#### Molecular docking

**FIGURE 7:**
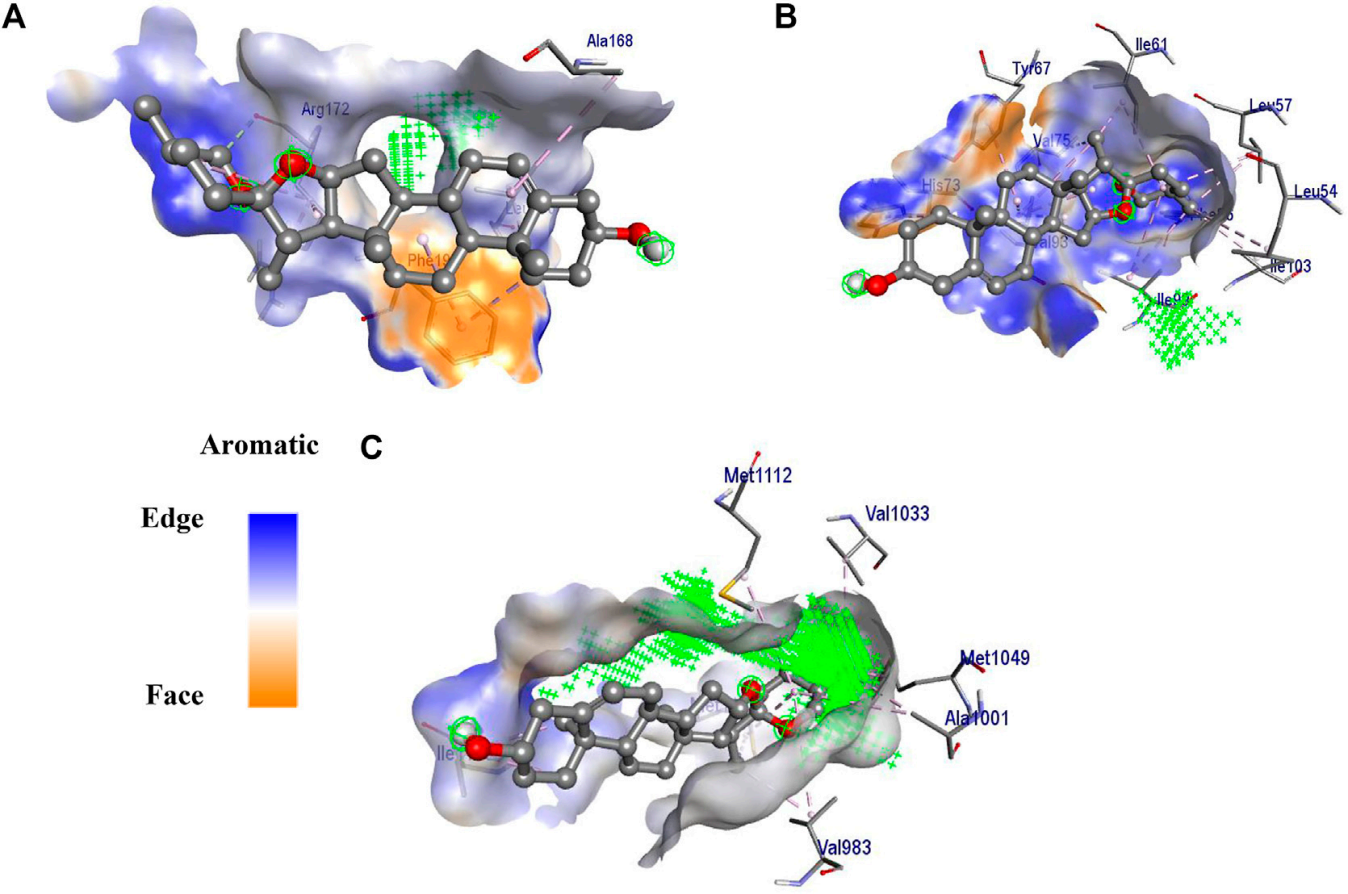
3D interaction of diosgenin with **(A)** *SRC*, **(B)** *MDM2* and **(C)** *IGF1R*. The surface around the ligand presents an aromatic surface. The ligand was presented in ball and stick. Amino acid residues were presented on a stick and the active site of the protein was presented with 
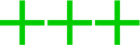
.

Among the three studied targets, diosgenin was predicted to possess a maximum binding affinity with *IGF1R* (ΔG = −8.6 kcal/mol, 11 alkyl interactions with *Val983*, *Met1126*, *Val1033*, *Met1112*, *Met1049*, *Ala1001*, and *Ile1130*) compared to *MDM2* (ΔG = -8.5 kcal/mol, 16 alkyl interactions with *Ile61*, *Val75*, *Phe86*, *Ile99*, *Leu54*, *Ile103*, *Leu57*, *Val93*, *Tyr67*, and *His73* and three van der Walls interactions with *Phe91*, *Gly58*, and *His96*) and *SRC* (ΔG = -7.4 kcal/mol, five alkyl interactions with *Phe194*, *Leu200*, and *Ala168*, four van der Waals interaction and three carbon-hydrogen bond with *Arg172*); Figure 7.

#### Molecular dynamics simulation

##### Stability of diosgenin–*IGF1R* complex

**FIGURE 8:**
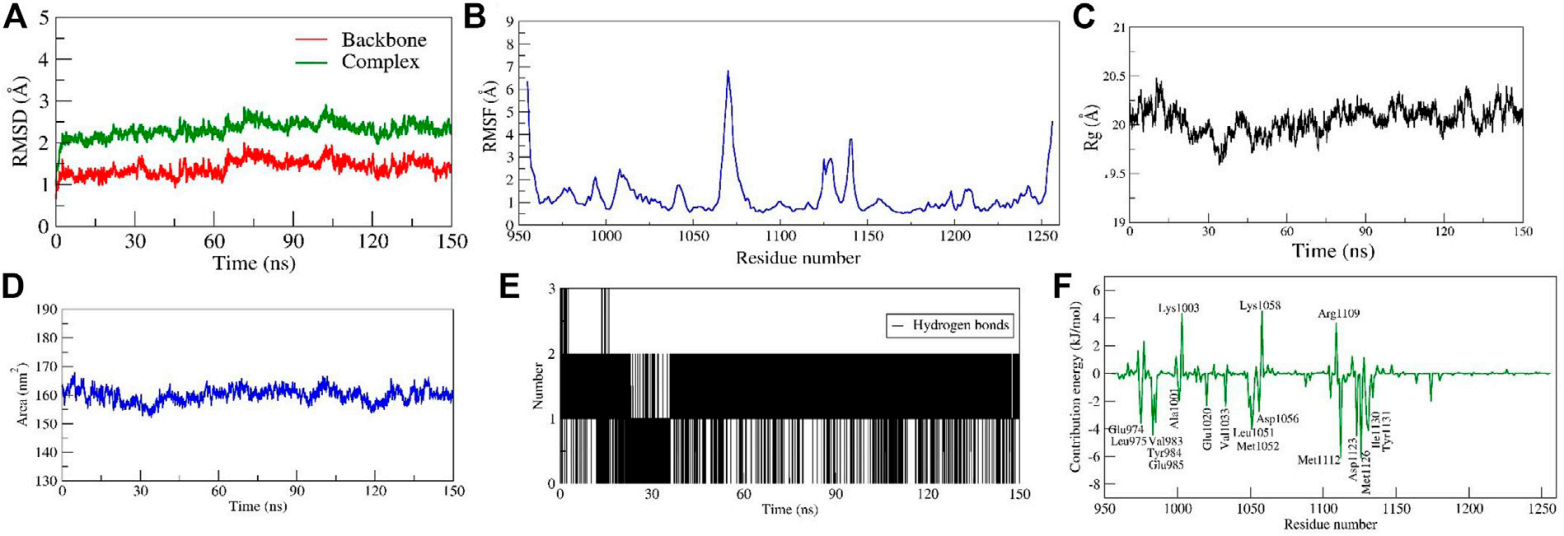
Parameters representing the structural stability of the Diosgenin-IGF1R complex. **(A)** Backbone and complex RMSD, **(B)** RMSF, **(C)** Rg, **(D)** SASA, **(E)** number of H-bond interactions, and **(F)** contribution energy plot demonstrates the significance of the ligand binding domain residues in stable complex formation.

The diosgenin-*IGF1R* complex showed stable dynamics up to 150 ns after a 20 ns equilibration phase. Initial backbone and complex RMSD values climbed steadily increased from 1.0 Å to 3.1 Å and ∼1.4 Å to ∼3.7 Å, respectively from 0 to 20 ns After 20 ns, it was discovered that the backbone and complex RMSD (∼2.5 Å and 3.0 Å, respectively) were stabilized with lesser fluctuations (Figure 8A). The loop-forming residues *Leu1064* to *Pro1077* showed comparatively greater fluctuations (7.0 Å). On the other hand, residues *Leu975*, *Val983*, *Ala1001*, *Glu1050*, *Asp1123*, and *Ile1130* that interacted with diosgenin during docking studies didn’t exhibit fluctuation because they were involved in stable non-bonded interactions. Additionally, it was discovered that residues *Gly1122* to *Tyr1131* forming the loop region were involved in ligand binding and showed the least RMS fluctuation (∼2.0 Å) (Figure 8B, Supplementary Movie S1). There was a formation of a compact globular shape, which was supported by a gradual drop in R*g* value from 20.5 Å to 19.6 Å and was further found to be stable at 20.0 Å (Figure 8C). The initial and final surface area occupied by *IGF1R* and diosgenin docked complex was 163.825 nm^2^ and 159.1106 nm^2^. The complex typically occupied 159.95 nm^2^ (Figure 8D). The complex formed 3 H-bonds of which two were consistent during the simulation (Figure 8E). It was discovered that diosgenin and *IGF1R* had relative binding energy of −35.143 ± 3.03 kcal/mol. Supplementary Table S2 summarizes the free energy contribution of diosgenin with *IGF1R*, *MDM2*, and *SRC*. The per residue contribution energy revealed that 16 residues from the binding pocket i.e. *Glu974*, *Leu975, Val983, Tyr984, Glu985, Ala1001, Glu1020, Val1033, Leu1051, Met1052, Asp1056, Met1112, Asp1123, Met1126, Ile1130,* and *Tyr1131* significantly contributed to the formation of a stable complex. These residues also scored the least per residue decomposition/contribution energy which ranged from −2.0 kJ/mol to −6.8 kJ/mol whereas the positive contribution energy of 4.0 kJ/mol was achieved by residues *Lys1003*, *Lys1058*, and *Arg1109* (Figure 8F).

##### Stability of diosgenin–*MDM2* complex

**FIGURE 9:**
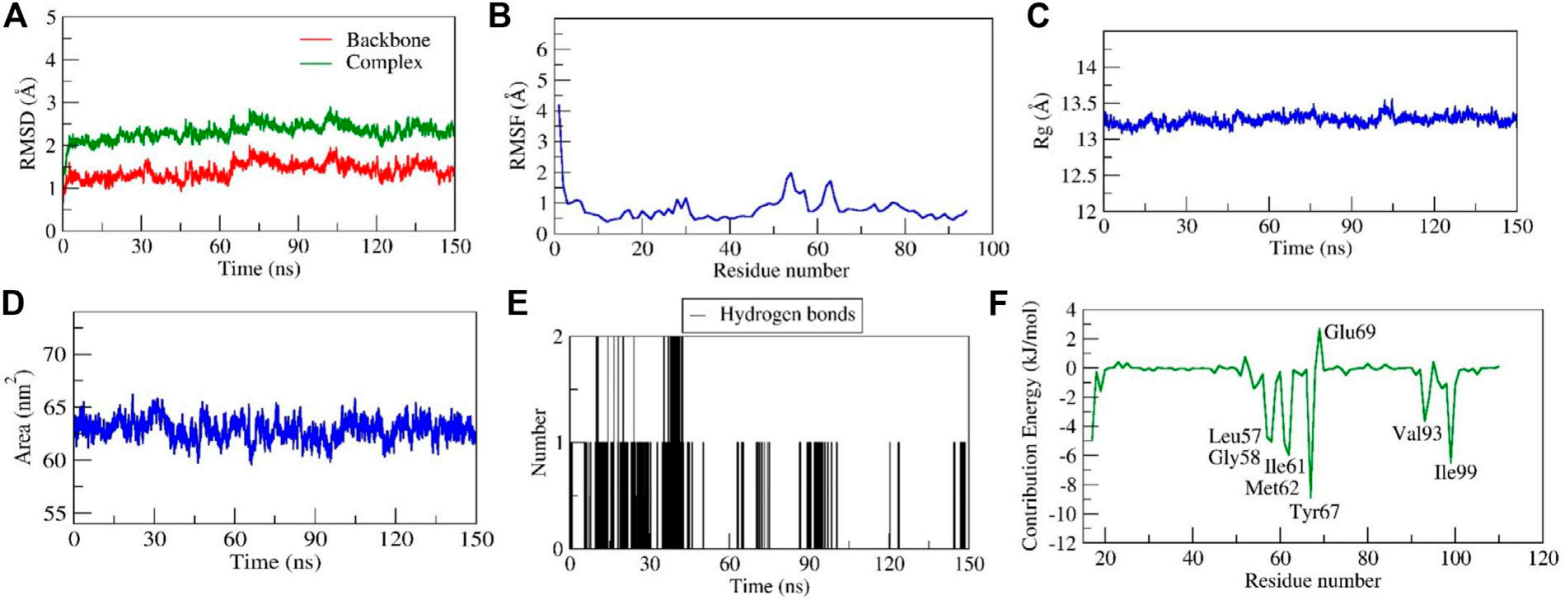
Parameters representing the structural stability of the Diosgenin-MDM2 complex. **(A)** Backbone and complex RMSD, **(B)** RMSF, **(C)** Rg, **(D)** SASA, **(E)** number of H-bond interactions, and **(F)** contribution energy plot demonstrates the significance of the ligand binding domain residues in stable complex formation.

Throughout a 150 ns production run, the Diosgenin-*MDM2* complex exhibited stable dynamics. The initial and final backbone RMSD values were 0.62 and 1.21, respectively, and an average was 1.40 Å. Similar to this, the initial and final RMSD of the docked complex *MDM2* and diosgenin were 0.92 and 2.13 respectively with an average of ∼2.30 Å (Figure 9A). The loop forming *Met17* to *Ser22* residues at the N-terminus had substantially greater fluctuations (∼4.2 Å). Further, residues interacting with diosgenin during docking studies (*Leu57*, *Tyr67*, *Phe91*, *Val93*, and *Ile99*) did not exhibit variations as they were involved in stable non-bonded interactions (Figure 9B and Supplementary Movie S2). By monitoring a stable Rg, a more compact and stable complex was formed. The complex’s initial and final Rg values were determined to be 13.2 Å and 1.31 Å. Similarly, the initial and final surface area occupied by *IGF1R* and diosgenin docked complex was 63.90 nm^2^ and 61.57 nm^2^. The complex had an average surface area of 62.89 nm^2^ (Figure 9D). Two H-bonds were established by the complex of which one was consistent during MD simulation (Figure 9E). The relative binding energy between diosgenin and *IGF1R* was discovered to be −34.619 ± 2.81 kcal/mol. Further, seven residues (*Leu57*, *Gly58*, *Ile61*, *Met62, Tyr67, Val93*, and *Ile99*) from the binding pocket scored the lowest per residue decomposition/contribution energy, ranging from −3.65 kJ/mol to −8.87 kJ/mol. These residues contributed significantly to forming the stable complex according to the per residue decomposition/contribution energy. The residue *Glu69* had positive contribution energy of 2.68 kJ/mol (Figure 9F).

##### Stability of diosgenin–*SRC* complex

**FIGURE 10:**
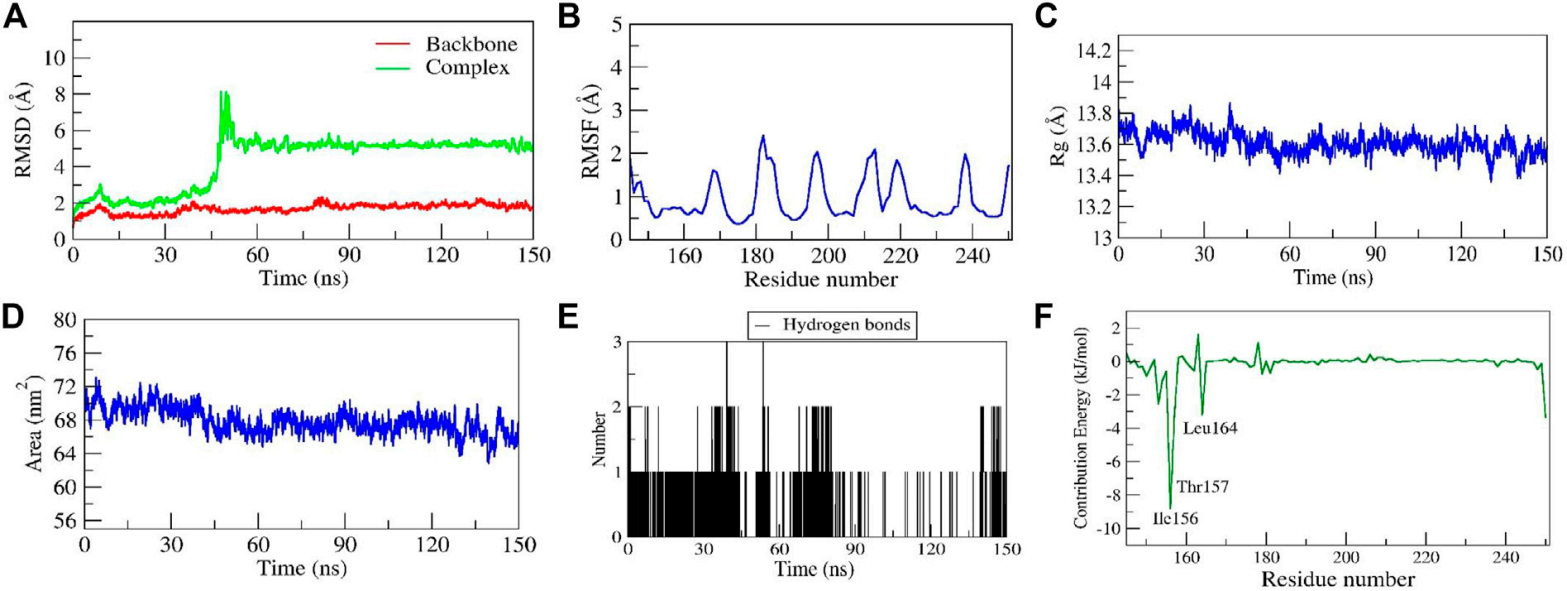
Parameters representing the structural stability of the Diosgenin-SRC complex. **(A)** Backbone and complex RMSD, **(B)** RMSF, **(C)** Rg, **(D)** SASA, **(E)** number of H-bond interactions, and **(F)** contribution energy plot demonstrates the significance of the ligand binding domain residues in stable complex formation.

Another diosgenin–*SRC* complex also showed stable dynamics with the RMSD value of < 2Å after the equilibration period of 50 ns(Figure 10A). In addition, the RMSD of the complex showed a sharp increase in its values after 40 ns to 8 Å (Figure 10A). The careful observation of the entire complex trajectory and representative snapshots extracted from the region reveal the structural transition of diosgenin from its primary binding pocket to the neighboring alternate pocket which formed a stable complex during the entire simulation period. Thus, for the first time, we report the existence of an alternate binding site other than the primary binding site on *SRC*. From the simulation movie (Supplementary Movie S3) it was observed that the complex undergoes major conformational changes in the secondary structure of the protein including the primary binding pocket, leading to the increased conformational flexibility of *SRC*, hence the diosgenin switches its original position from the primary binding pocket to the reported alternate binding pocket (formed by the residues *Tyr152* to *Leu164*). The residual fluctuations observed show fewer C-alpha fluctuations (<2.2Å), interestingly the residues from the primary binding pocket as well as new reported alternate binding pocket show the least RMSF values ∼0.5Å (Figure 10B), mainly due to the stable non-bonded interactions shown by them. The complex diosgenin–*SRC* gains a compact globular shape during the simulation, thus we propose the stable complex formation was favored after the equilibration period of MD simulation (Figure 10C). The solvent-accessible surface area represents an exposure of the hydrophobic residues to the solvent, here in this complex SASA exhibited a similar trend as that of Rg values. SASA value decreased gradually till the equilibration state and further, it stabilized till the simulation end (Figure 10D), suggesting the proper folding of the hydrophobic core including both the binding sites. Two H-bonds were established by the complex of which one was consistent during MD simulation (Figure 10E). The relative binding energy between diosgenin to *SRC* was discovered to be −17.994 ± 5.67 kcal/mol. In addition, three residues *i.e., Ile156*, *Thr157*, and *Leu164* from the binding pocket scored the lowest energy contribution per residue. These residues contributed significantly to forming the stable complex according to the per residue decomposition/contribution energy. The residue *Ile156* had the least contribution energy of −8.9 kJ/mol (Figure 10F).

#### Principal component and dynamic cross-correlation matrix

**FIGURE 11:**
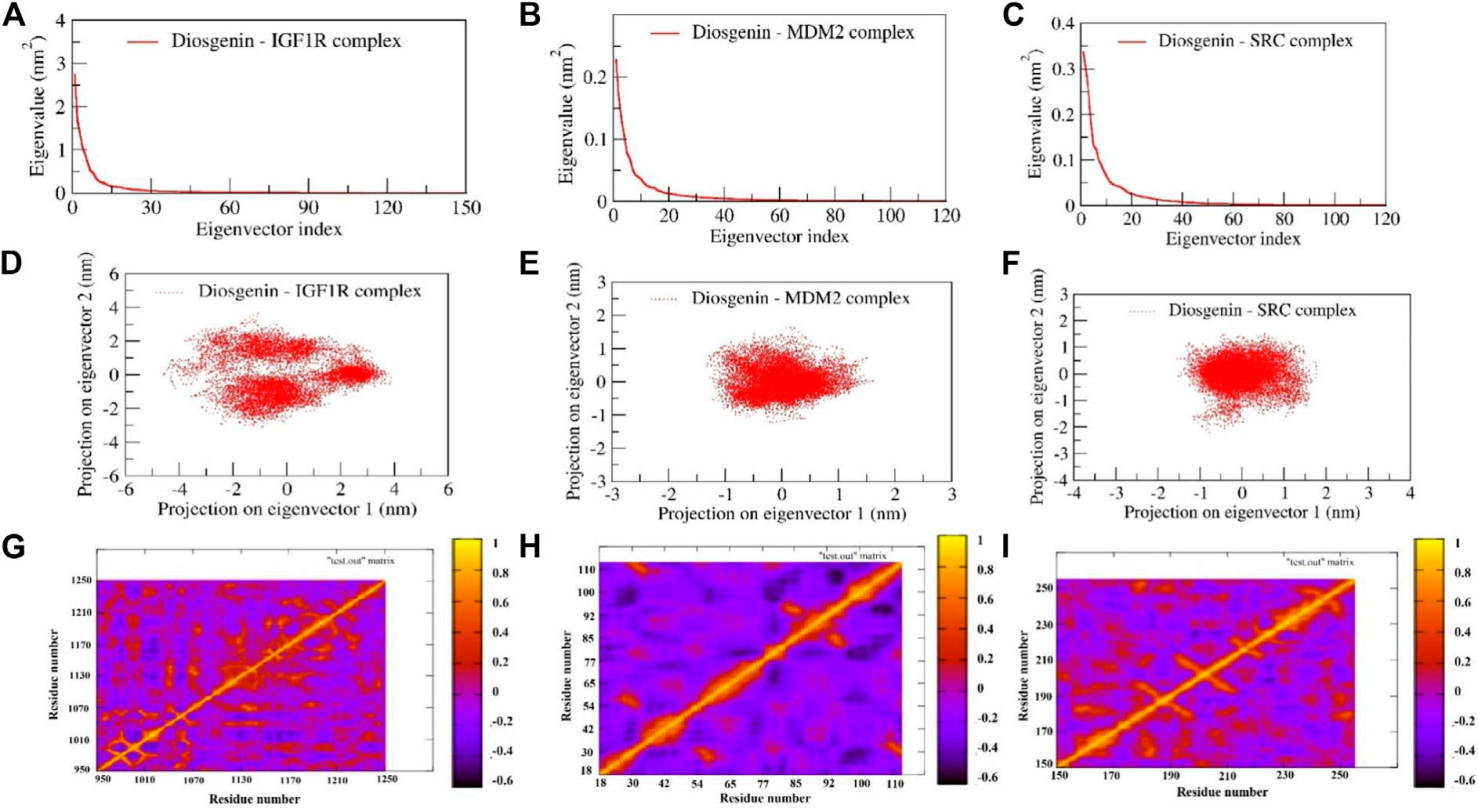
Represents the principal component analysis and dynamic cross-correlation matrix of complexes: The first 120 eigenvectors were plotted v/s eigenvalues for diosgenin with **(A)** *IGF1R* **(B)** *MDM2*, and **(C)** *SRC*. The collective motions of diosgenin with **(D)** *IGF1R* **(E)** *MDM2*, and **(F)** *SRC* using projections of MD trajectories on two eigenvectors corresponding to the first two principal components. Dynamic cross-correlation matrix of Cα atoms observed in diosgenin with **(G)** *IGF1R* **(H)** *MDM2*, and **(I)** *SRC*. The amber-colored positive regions reflect strongly correlated Cα atom movements (C*ij* = 1), whereas the blue-colored negative regions show anticorrelated motions (C*ij* = -1).

PCA is a statistical technique being used to study the dynamics of bimolecular complexes as it limits the 3 N (N = number of atoms in the protein) degrees of freedom describing functionally crucial motions of the protein. It was observed that in all the complexes maximum dynamics during the simulation have been captured by the first 10 eigenvectors, of which the first two contributed significantly to the collective motions exerted by all the simulated complexes (Figures 14A–C). Hence, we examined the collective motion sampled by the first two principal components (PCs), and 2D projections for PC1 and PC2 were plotted (Figures 11D–F). The complexes of diosgenin with *MDM2* and *SRC* express the compact clusters in the conformational spaces those range from -1.5 to 1.5. In the MD trajectory of complexes Diosgenin-*SRC* and Diosgenin-*MDM2*, PC1 and PC2 (top two modes) showed the uniform distribution across the configurational space while the remaining complex of Diosgenin-*IGF1R* showed a large diversity in the conformational space and was widely clustered in the range of -4.5 to 4.5 (Figure 11D). MD trajectory sampled three states of the protein as seen by the three individual clusters in the scatterplot of PC1 v/s PC2. Herein, we propose that the Diosgenin-*IGF1R* complex has undergone significant conformational changes in the secondary structure during the simulation those favored in forming a stable complex. However, other complexes namely Diosgenin-*SRC* and Diosgenin-*MDM2* were well stabilized and undergone comparatively lesser conformational changes in the secondary structure hence exhibited compact cluster in the conformations space. Further, the convergence of sampling was also analyzed by calculating the cosine content of all the trajectories obtained. The cosine content was calculated for the trajectories of the first top two principal components (PC1 and PC2) for complexes Diosgenin-*SRC* and Diosgenin-*MDM2*, and Diosgenin-*IGF1R* is observed as 0.024225, 0.0175249 and 0.158163 respectively. It has been reported that the value of cosine content for the first few PCs close to 1 indicated bad sampling of the trajectory pointing to all our simulation trajectories being well converged and properly sampled in the free energy landscape.

The concerted motion exerted by the three complexes during the simulation was examined using DCCM (dynamic cross-correlation matrix). The calculation of the correlation matrix is utilized to depict the dynamical information of proteins in two dimensions. To observe the correlation in the dynamics of the binding site correlation matrix over the stable trajectory of all the complexes were plotted (Figures 11F,G). The diagonal orange-red line indicates the self-correlation of the individual residues with themselves. The orange-red region in the correlation map signifies the concerted movement of the residues in the same direction whereas the dark blue region represents anti-correlated fluctuations. The N- and C-terminal region of the complex Diosgenin-*MDM2* represents strong anticooperative movement with each other. Comparatively, this complex exhibited a maximum amplitude of negative correlation across all the residues. The maximum region in the complex Diosgenin-*IGF1R* showed positive correlation mainly at the binding pocket region (residues *Glu974, Leu975, Val983, Tyr984, Glu985, Ala1001, Glu1020, Val1033, Leu1051, Met1052, Asp1056, Met1112, Asp1123, Met1126, Ile1130,* and *Tyr1131*). This signifies the closure movement observed at the binding pocket region facilitating stable complex formation (Supplementary Movie S1). Similarly, in the Diosgenin-*SRC* complex, the binding pocket residues (*Arg158*, *Glu181*, *Thr182*, *Cys188*, *Lys203*-*Lys206*) showed moderately positive cooperative motion compared to other regions in the *SRC* structures. Interestingly, the residues from the alternate binding region show the cooperative motion results in stable non-bonded contact with the newly reported alternate binding pocket.

In general, the conformational flexibility of all the complexes *i.e.,* Diosgenin-*IGF1R*, Diosgenin-*MDM2*, and Diosgenin-*SRC* varies greatly as observed in the DCCM plot and the collective dynamics nature observed in the PCA and DCCM plot favors the stable complex formation during the simulation.

#### Survival analysis of hub genes

To evaluate the prognostic significance of genes, we analyzed the survival curves of each gene. Among 21 genes, 17 genes were observed to had the significant effect over the Kaplan–Meier survival analysis *i.e., APP* (log-rank *p* = 0.0018), *AR* (log-rank *p* = 0.0033), *CDK4* (log-rank *p* = 0.0062), *CYP19A1* (log-rank *p* = 0.036), *FGFR2* (log-rank *p* = 2.9 × 10^–5^), *GRM1* (log-rank *p* = 0.027), *IGF1R* (log-rank *p* = 3.2 × 10^–05^), *LYN* (log-rank *p* = 0.017), *MDM2* (log-rank *p* = 0.0012), *MDM4* (log-rank *p* = 0.044), *NR3C1* (log-rank *p* = 0.00038), *PDGFRB* (log-rank *p* = 1.2 × 10^–06^), *PRCP* (log-rank *p* = 0.015), *PTPN1* (log-rank *p* = 0.00041), *RET* (log-rank *p* = 0.03), *SRC* (log-rank *p* = 0.0039), and *STAT3* (log-rank *p* = 1.4 × 10^–06^). Similarly, four genes *i.e., CRHR1* (log-rank *p* = 0.09), *CYP17A1* (log-rank *p* = 0.14), *CYP3A4* (log-rank *p* = 0.056), and *FASN* (log-rank *p* = 0.15) were insignificantly linked with disease prognosis; Table S3.

#### Gene expression analysis in tumor, normal and metastatic tissues

**FIGURE 12:**
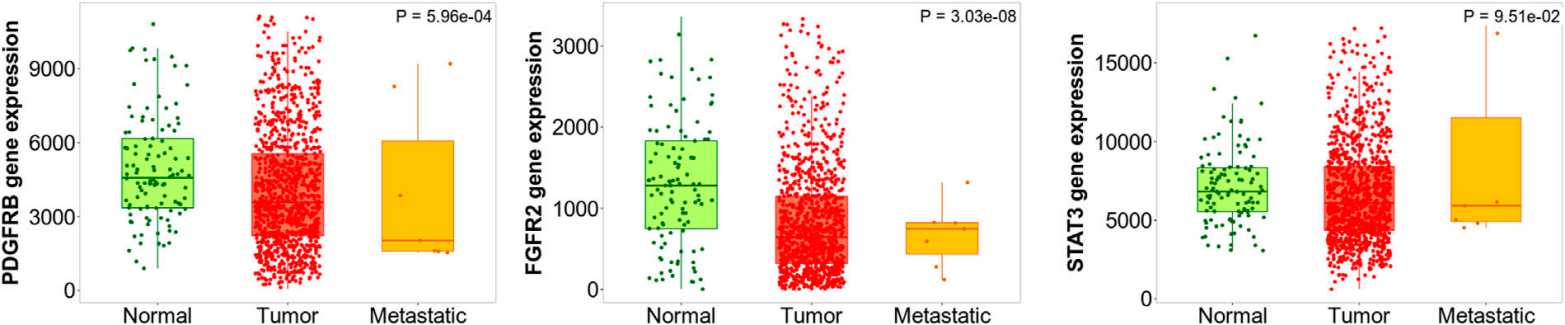
Differential gene expression analysis of *PDGFRB*, *FBFR2*, and *STAT3* in tumor, normal and metastatic tissues. These genes were identified to possess the least log-rank test.

Kruskal Wallis test revealed the significant expression (*p* = 5.96e-04) of the *PDGFRB* gene in breast invasive carcinoma compared to normal and metastatic samples. In addition, the similar observations were also noted for the *FGFR2* (*p* = 3.03e-08) and *STAT3* (*p* = 9.51e-02) genes. In addition, Dunn test pointed the difference in *PDGFRB* expression in the normal vs*.* tumor (*p* = 7.74e-05), tumor vs*.* metastasis (*p* = 5.66e-02), normal vs*.* metastasis (*p* = 2.61e-01), *FGFR2* in normal vs*.* tumor (*p* = 2.31e-04), tumor vs*.* metastasis (*p* = 2.98e-02), and normal vs*.* metastasis (*p* = 3.41e-01) and *STAT3* normal vs*.* tumor (*p* = 1.59e-02), tumor vs*.* metastasis (*p* = 4.26e-01), and normal vs*.* metastasis (*p* = 3.57e-01); Figure 12).

**FIGURE 13:**
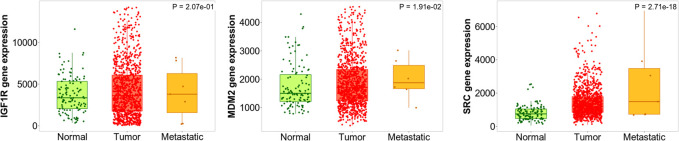
Differential gene expression analysis of *IGF1R*, *MDM2*, and *RSC* in tumor, normal and metastatic tissues. Diosgenin was predicted to possess the highest binding affinity with these targets.

Among *IGF1R*, *SRC*, and *MDM2*, all the genes had a significant difference in gene expression compared to three different tissues *i.e., IGF1R* (*p* = 2.07e-01), *SRC* (*p* = 2.71e-18), and *MDM2* (*p* = 1.91e-02) which were evaluated using Kruskal Wallis test. In addition, there was a significant difference in *MDM2* expression in normal vs*.* tumor (*p* = 3.83e-03), tumor vs*.* metastasis (6.87e-02), and normal vs*.* metastasis (2.03e-01) as revealed by the Dunn test. Likewise, there was a significant difference in *SRC* expression in normal vs*.* tumor (*p* = 1.72e-19), tumor vs*.* metastasis (1.92e-03), and normal vs*.* metastasis (2.62e-01). Likewise, there was a significant difference in *IGF1R* expression in normal vs*.* tumor (*p* = 4.25e-02), tumor vs*.* metastasis (*p* = 4.91e-01), and normal vs*.* metastasis (*p* = 3.19e-01); Figure 13.

### *In vitro* pharmacology

#### BSL bioassay

Exposure to the different concentrations of diosgenin and doxorubicin showed concentration-dependent brine shrimp lethality. In addition, the LC_50_ was found to be 19.15 and 0.71 μg/ml respectively reflecting the doxorubicin to be 27.06 times more potent than diosgenin (Supplementary Figure S4).

#### *In vitro* MTT assay

Here, the MTT assay showed the IC_50_ of the diosgenin to be significantly higher than that of the doxorubicin in MCF7 (*p* < 0.001), MDA-MB-231 (*p* < 0.05), SKBR3 (*p* < 0.05) and Vero (*p* < 0.001) compared to the doxorubicin. *In vitro* MTT assay on MCF7 cell lines reflected the doxorubicin (IC_50_ 3.21 ± 0.29 μg/ml) to be 3.7 times more potent than the diosgenin (IC_50_ 12.05 ± 1.33 μg/ml). In addition, over the MDA-MB-231 cell lines, the IC_50_ of diosgenin was found to be (45.54 ± 23.41) µg/ml compared to doxorubicin (6.30 ± 2.67) µg/ml which suggests the doxorubicin to be 7.2 times more potent than diosgenin. In addition, the diosgenin and doxorubicin had the IC_50_ (15.11 ± 5.32) µg/ml and (5.47 ± 1.09) µg/ml respectively over SKBR3 reflecting the doxorubicin to be 2.76 times more potent than that of diosgenin. In addition, the IC_50_ of diosgenin and doxorubicin was found to be (17.78 ± 7.86) and (8.18 ± 8.18) µg/ml respectively over the T47D cell lines to point doxorubicin to be 2.17 times more potent. Further, diosgenin and doxorubicin had the IC_50_ of (38.59 ± 4.03) and (7.05 ± 0.69) µg/ml respectively in which the doxorubicin was found to be 7 times more cytotoxic than Vero cell lines; Supplementary Figure S5; Supplementary Table S4.

#### *In vitro* scratch assay

After the 72-h exposure to different cells, it was observed that diosgenin (200 μg/ml) had the highest effect on SKBR3 cell lines (18.22 ± 1.237%) to prevent cell migration compared to the rest. In addition, we observed a significant difference in percentage scratch closure within MCF vs*.* SKBR3 (*p* < 0.01), MDA-MB-231 vs*.* SKBR3 (*p* < 0.05), SKBR3 vs*.* T47D (*p* < 0.01) and SKBR3 vs*.* Vero (*p* < 0.001) cell lines (Supplementary Figure S6). In addition, the concentration and time-dependent effect of the diosgenin on scratch closure are presented in Supplementary Figure S7.

#### Effect of diosgenin and doxorubicin over H_2_O_2_-induced stress in cell lines

Upon the 24 h exposure of the H_2_O_2_, it was observed that diosgenin has an equivalent effect to ascorbic acid over MCF cell lines with an IC_50_ (7.68 ± 0.51) µg/ml and (7.13 ± 0.31) µg/ml respectively. In addition, diosgenin and ascorbic acid showed the IC_50_ of (13.58 ± 0.90) µg/ml and (10.60 ± 1.60) µg/ml respectively over the MDA-MB-231 cell lines. However, diosgenin (IC_50_ 6.68 ± 0.67 μg/ml) had more effect over the SKBR3 compared to ascorbic acid (9.39 ± 3.09 μg/ml). Similarly, diosgenin and doxorubicin had the IC_50_ (8.90 ± 0.98) and (9.14 ± 0.78) µg/ml respectively over T47D cell lines. Further, the diosgenin and ascorbic acid had the IC_50_ (13.72 ± 1.83) µg/ml and (12.68 ± 4.53) µg/ml respectively over the Vero cell lines (Supplementary Table S5). The concentration-dependent effect of diosgenin and ascorbic acid is presented in Supplementary Figure S8.

#### Effect of diosgenin on Warburg effect

It was observed that diosgenin had efficacy in dealing glucose uptake in tumor cells. The EC_50_ of the diosgenin was observed to be significantly higher in glucose uptake in MCF7 (*p* < 0.001), SKBR3 (*p* < 0.05), T47D (*p* < 0.001), and Vero (*p* < 0.01) cell lines compared to metformin in the presence of insulin. It was observed that diosgenin had a comparatively higher EC_50_ (26.19 ± 2.77) µg/ml compared to metformin (3.10 ± 0.99) µg/ml which showed the diosgenin to possess a comparatively lower glucose uptake efficacy than metformin in MCF7 cell lines. In addition, in MDA-MB-231 cell lines, diosgenin and metformin had an equivalent EC_50_ in promoting glucose uptake *i.e.,* EC_50_ (17.92 ± 1.19) and (17.03 ± 2.59) µg/ml respectively. Furthermore, it was observed that diosgenin had comparatively higher the EC_50_ in promoting the glucose uptake in SKBR3 (EC_50_ 19.99 ± 2.91 μg/ml), T47D (EC_50_ 37.47 ± 1.75 μg/ml), and Vero (EC_50_ 15.27 ± 0.95 μg/ml) compared to metformin (EC_50_ SKBR3; 11.74 ± 3.19 μg/ml, T47D; 12.50 ± 1.42 μg/ml, and Vero; 11.79 ± 1.65 μg/ml); Supplementary Table S6; Supplementary Figure S9

## Discussion

The present study focused on tracing the targets of diosgenin and evaluates the probably triggered pathways *via* the PPI in breast cancer. In this regard initially, we predicted the targets using three open-source platforms *i.e.* SwissTargetPrediction, DIGEP-Pred, and BindingDB; matched with estrogen receptor-positive breast cancer targets (UMLS CUI: *C2938924*); retrieved from DisGeNET in which diosgenin was predicted to target 21 different proteins *i.e. APP*, *CDK4*, *CRHR1*, *CYP17A1*, *CYP19A1*, *CYP3A4*, *FASN*, *FGFR2*, *GRM1*, *IGF1R*, *LYN*, *MDM2*, *MDM4*, *PDGFRB*, *PRCP*, *PTPN1*, *RET*, and *SRC*, (predicted in SwissTargetPrediction) *AR* and *NR3C1* (predicted in DIGEP-Pred), and *STAT3* (predicted in SwissTargetPrediction and BindingDB) in the breast cancer. Herein concerning the KEGG database, it was observed that 36 different pathways were triggered in which 12 pathways *i.e.* pathways in cancer; *hsa05200*, prostate cancer; *hsa05215*, viral carcinogenesis; *hsa05203*, glioma; *hsa05214*, melanoma; *hsa05218*, bladder cancer; *hsa05219*, MicroRNAs in cancer; *hsa05206*, Kaposi sarcoma-associated herpesvirus infection; *hsa05167*, proteoglycans in cancer; *hsa05205*, central carbon metabolism in cancer; *hsa05230*, non-small cell lung cancer; *hsa05223*, and pancreatic cancer; *hsa05212* were identified which explores the anti-cancer pharmacological spectra of the diosgenin.

Breast cancer is one of the main causes of death among women. Chiefly, the cells lining the milk-forming duct of the mammary glands are the origination of breast cancer (Herbein and Kumar, 2014) which can be further subdivided based on the presence or absence of the hormone receptors *i.e.,* estrogen and progesterone subtypes and human epidermal growth factor receptor-2 (*HER2*). In addition, the estrogen receptor pathway triggers hormone receptor-positive breast cancer (Rani et al., 2019). Similarly, in *HER2*-positive breast cancer, *HER2* triggers RAS/RAF/MAPK and PI3K/AKT signaling pathways that stimulate cell growth, survival, and differentiation (Dittrich et al., 2014). We discovered 21 diosgenin-regulated proteins that are involved with oestrogen receptor-positive breast cancer (UMLS CUI: *C2938924*) targets in this investigation. In addition, three pathways *i.e.,* PI3K-Akt (modulated five genes *i.e., CDK4*, *MDM2*, *PDGFRB*, *IGF1R*, and *FGFR2*), Ras (modulated three genes *i.e., PDGFRB*, *IGF1R*, and *FGFR2*), and MAPK (modulated three genes *i.e., PDGFRB*, *IGF1R*, and *FGFR2*) signaling pathways associated to *HER2* positive breast cancer were modulated. Since diosgenin modulated three pathways closely associated with *HER2*-linked pathogenesis, it can be speculated that diosgenin could act *via* the manipulation of *HER2*.

It has previously been proposed that Akt activation influences endocrine resistance in metastatic breast cancer. In addition, Akt activation in the downstream pathway of *HER2* could resist the endocrine therapy of breast cancer (Tokunaga et al., 2006). Furthermore, Ras proteins activate the cytoplasm and extracellular signaling networks *via* receptor tyrosine kinase and are involved in cell proliferation, survival, growth, metabolism, motility, and apoptosis, and their hyperactivation promotes the growth and progression of breast cancer. In addition, Ras’s intracellular localization, activation, and signaling have been used in developing therapeutic candidates against breast cancer *via* the enzymes involved in posttranslational modification of Ras *e.g.,* farnesyltransferase and geranylgeranyltransferase 1 (Moon, 2021). Further, *MAPK* links the extracellular mitogenic signals to cell proliferation which may be concerned with or act independently towards estrogen-mediated events in breast cancer cells (Yue et al., 2002). In the present study, we identified the modulation of the above-modulated pathways *i.e.,* PI3K-Akt signaling pathway (false discovery rate: 0.0011, strength: 1.12), Ras signaling pathway (false discovery rate: 0.0263, strength: 1.09), and MAPK signaling pathway (false discovery rate: 0.0446, strength: 0.99). This suggests the probability of involvement of these pathways linked to *HER2* function which could be modulated by diosgenin in breast cancer.

The role of *EGFR* dysregulation or mutation in cancer etiology, particularly breast cancer, has been proposed previously. However, resistance towards *EGFR* tyrosine kinase inhibitors may occur due to secondary mutations (T790M), activation of secondary pathways (AXL, c-Met, HGF), aberrant downstream pathways (K-RAS mutations, loss of *PTEN*), deregulation of the *EGFR* tyrosine kinase-mediated apoptosis, histological transformation, and ATP binding cassette transporter effusion, etc (Huang and Fu 2015).

The FoxO signaling system interacts with the PI3K-Akt signaling pathway and relates to cancer progression, particularly breast cancer advancement (Farhan et al., 2017). In addition, FoxO negatively regulates activated *EGFR* signaling which was demonstrated *via* the *in vitro* cell line culture method and *in vivo* models (Sangodkar et al., 2012). Here, in the present study, we identified the regulation of the FoxO signaling pathway *via* the modulation of four genes *i.e. MDM2*, *STAT3*, *IGF1R*, and *GRM1* which could support the functioning of the PI3K-Akt and *EGFR* signal against breast cancer pathogenesis.

Furthermore, the Rap1 signal has been linked to tumor cell proliferation, invasion, and metastasis through regulating integrin- or cadherin-mediated cell function, cytoskeletal alterations, protease (metal metalloprotease) production, and cell adhesion (Zhang et al., 2017). In addition, Rap1 has been traced to attenuate metastasis and *EGFR*-triggered carcinoma (Huang et al., 2012). Since breast cancer progression is closely linked to *EGFR* tyrosine kinase signal; it can be speculated that diosgenin-mediated Rap1 signal could attenuate tumor invasion and metastasis; was observed to be modulated *via* the modulation of three genes *i.e., PDGFRB*, *IGF1R*, and *FGFR2*.

The p53 acts as a transcription factor for p21 *via* cyclin-CDK interactions which is important for the transition of the G2 phase to the mitosis phase (Alam et al., 2015). In addition, p21 protects cells from apoptosis, regulates the cell cycle, causes apoptosis, and decreases cell proliferation in tumor cells (Wang et al., 2021). Mutant p53 has been pointed to as the guardian of the cancer cells (Mantovani et al., 2019) and is also associated with worsening breast cancer affecting overall survival (Gasco et al., 2002). In the present study, we identified the regulation of the three genes *i.e., CDK4*, *MDM2*, and *MDM4* which could activate the p53 signal against breast cancer. Furthermore, excessive plasma prolactin levels have been associated with an increased risk of breast cancer in both premenopausal and postmenopausal women; is more prominent in estrogen or progestogen receptor cancer type (Tworoger et al., 2004; Tworoger et al., 2006). Here, in the present study, we identified the diosgenin to regulate the prolactin signaling pathway *via* the regulation of three genes *i.e., STAT3*, *CYP17A1*, and *SRC* which could avoid estrogen or progestogen receptor-mediated breast cancer progression. In addition, chemokine signals are not limited to tissue differentiation, hematopoiesis, inflammation, and immune regulation but also process tumor development by triggering angiogenesis, metastasis, drug resistance, and immunity of breast cancer (Liu et al., 2020). In the present study, we identified the diosgenin to trigger the chemokine signaling pathway, and regulated three genes *i.e., STAT3*, *SRC*, and *LYN* at 1.18 strength and 0.0168 false discovery rate). In addition, in enrichment analysis *i.e.,* tissues, Pfam, InterPro, and compartments enrichment analysis we observed the multiple proteins that are concerned with the breast cancer prognosis.

Also, in the present study, we identified three proteins *i.e., IGF1R*, *MDM2*, and *SRC* in diosgenin-protein(s)-pathway(s) interaction. Hence, these were further considered for post-network analysis *i.e.,* molecular docking and molecular dynamics simulation. Apart from handling the transcription, *IGF1R* can trigger the growth and metastasis of malignant melanoma cells through the PI3K-Akt signaling pathway (Ekyalongo and Yee, 2017). Diosgenin was anticipated to interact with *IGF1R* in the current investigation, potentially preventing breast cancer metastasis and tumor invasion, which could be PI3K-Akt driven, as previously discussed.

The *IGF1R* is a transcription factor that binds to DNA and influence transcription. Both ERK1/2 and AKT are involved in the transcriptional control of the *IGF1R* gene. MicroRNA-139-5p modulates the growth and metastasis of malignant melanoma cells *via* the PI3K/AKT signaling pathway by binding to *IGF1R* (binding energy -8.6 kcal/mol). Previously, *MDM2* amplification has been reported to relate to estrogen receptor status and its presence has been indicated in human breast cancer cell (Quesnel et al., 1994); was observed to be manipulated with diosgenin in the third cluster of the PPI binding (binding energy −8.5 kcal/mol). Likewise, another modulated protein *i.e.,* SRC has been reported to increase its expression in human breast cancer by 4–30 fold which was evidenced *via* both immunohistochemistry and immunoblotting (Verbeek et al., 1996); was also predicted to be modulated by diosgenin interaction (binding energy -7.4 kcal/mol). In addition, since 17 genes i.e. *APP*, *AR*, *CDK4*, *CYP19A1*, *FGFR2*, *GRM1*, *IGF1R*, *LYN*, *MDM2*, *MDM4*, *NR3C1*, *PDGFRB*, *PRCP*, *PTPN1*, *RET*, *SRC*, and *STAT3* were observed to have a significant role in disease prognosis, it can be speculated that the above-modulated proteins are primarily concerned with breast cancer management with diosgenin treatment.

The docking study revealed diosgenin to interact with active site residues of three potential targets involved in breast cancer *via IGF1R, MDM2*, and *SRC*. Diosgenin formed stable intermolecular interactions throughout 150 ns MD simulation revealing them as the best lead. Among the interactions of diosgenin with *IGF1R*, diosgenin interactions with *Leu975, Val983, Met1112, Met1126*, and *Ile1130* were consistent in both docking and MD simulation. Multiple studies have demonstrated these residues involve the pocket as a primary binding site (also validated by PrankWeb server; https://prankweb.cz/) for inhibition of *IGF1R* (Munshi et al., 2002; Li et al., 2009; Guo et al., 2015). This indicates, that diosgenin as a potent lead hit against *IGF1R*. Similarly, diosgenin scored the lowest binding energy of -8.5 kcal/mol and binding free energy of -34.619 kcal/mol and formed stable interactions *Leu57*, *Gly58*, *Ile61*, *Met62*, *Tyr67*, *Val93*, and *Ile99* throughout the 150 ns MD production run. Both docking and MD simulation revealed diosgenin as a potent lead hit for targeting *MDM2*. Interestingly, a study by Li et al. (2021) identified AG-690/37072075 and AO-022/43452814 as potent anticancer lead hits against *MDM2*. These molecules were predicted to interact with the residues “*Leu57, Gly58, Ile61, Met62, Tyr67, Val93,* and *Ile99*” and were also found to inhibit p53-MDM2 interaction in wild-type p53 cells. Hence, we believe that diosgenin may interfere with the p53-*MDM2* interaction (*MDM2* inhibits the transcriptional activity of p53 by attaching to its transactivation domain), and further experimental studies are required to validate our findings. Further, diosgenin scored -7.4 kcal/mol binding energy against SRC and formed interaction with *Ala168*, *Arg172*, *Phe194*, and *Leu200*. The structural shift of diosgenin from its initial binding pocket to the neighboring alternative pocket was revealed through MD simulation, and the complex remained stable throughout the entire simulation. As a result, we are the first to disclose the discovery of a secondary binding site in the *SRC*. The simulation movie (Supplementary Movie S3) shows that the complex undergoes significant conformational changes in the protein’s secondary structure, including the primary binding pocket, increasing the conformational flexibility of *SRC* and causing diosgenin to switch positions from the primary binding pocket to the alternate binding pocket that has been reported. The PCA and DCCM revealed that diosgenin with *IGF1R*, *MDM2*, and *SRC* exhibited significant differences in conformational flexibility and also support the stable complex formation during the simulation.

**FIGURE 14:**
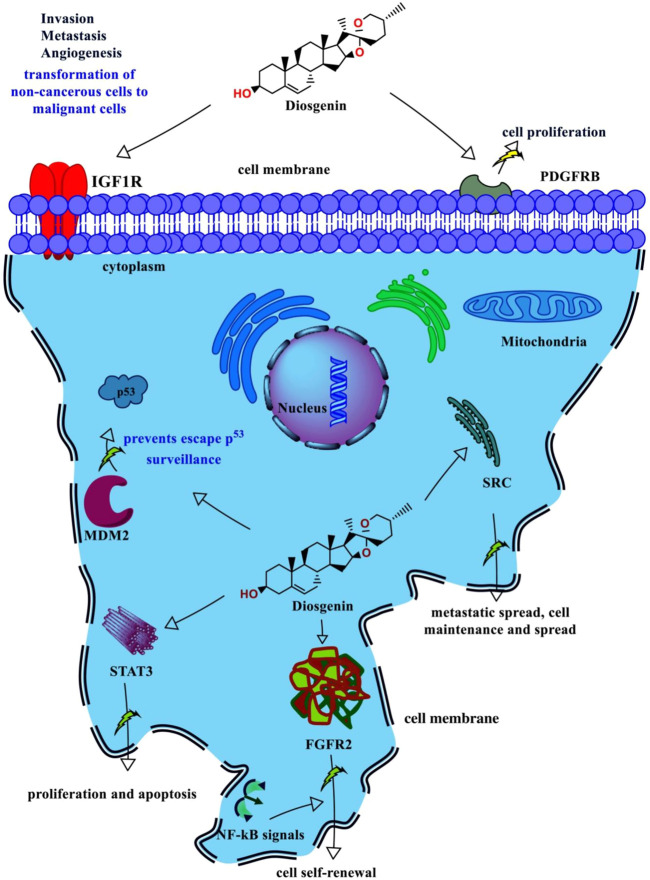
Mechanism of action of diosgenin against oestrogen receptor-positive breast cancer. Diosgenin acts on the two cell surface proteins *IGF1R* and *PDGFRB* and inhibits invasion, metastasis, angiogenesis, and cell proliferation. In addition, it acts on the four cytoplasmic proteins i.e., *MDM2*, *STAT3*, *FGFR2,* and *SRC*. Diosgenin may inhibit the cell to escape p53 surveillance by binding with *MDM2*, inhibit cell proliferation and promote apoptosis by binding to *STAT3*, interfere with *NF-kB* signals check the cancer cell self-renewal, and inhibit the metastatic spread by inhibiting the SRC action.

**FIGURE 15:**
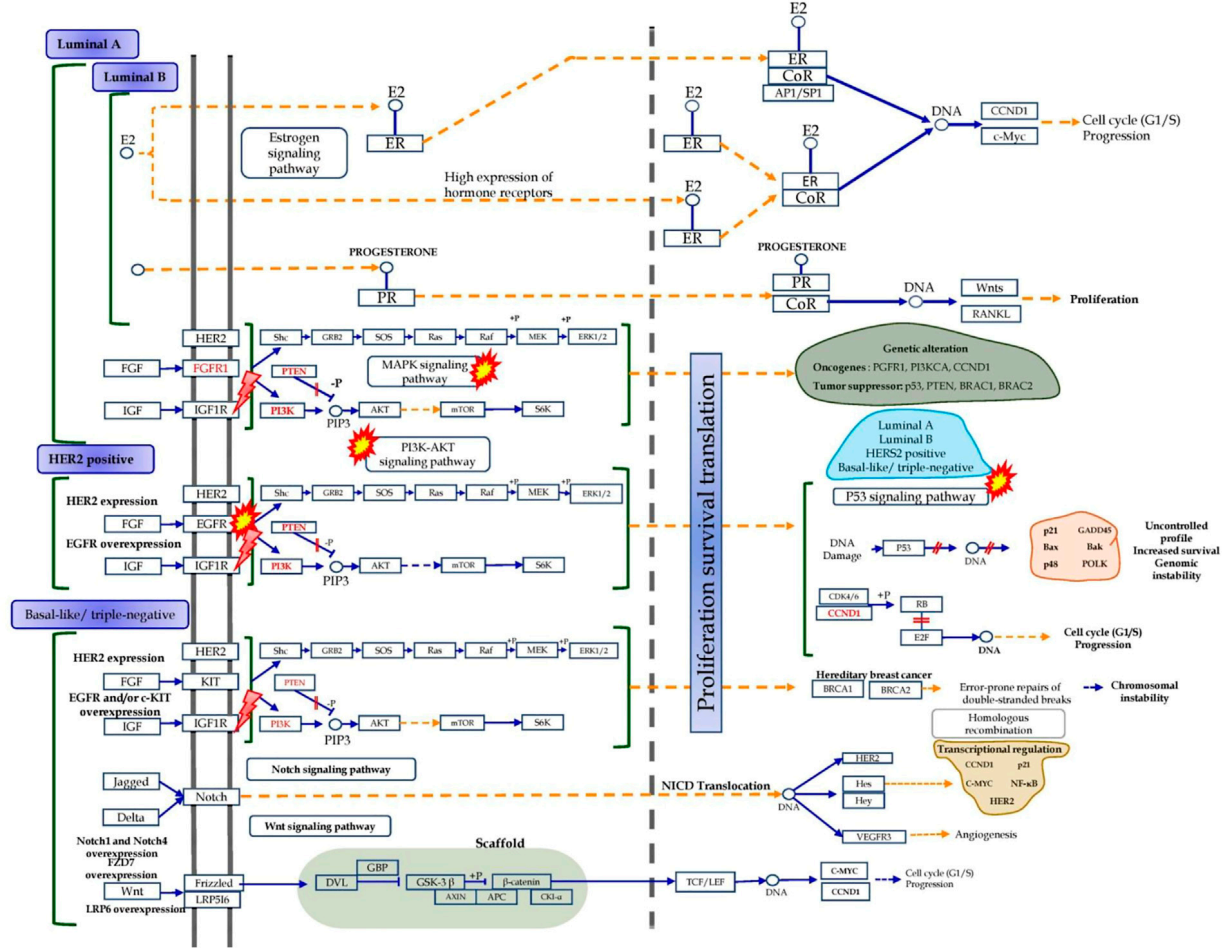
Probable checkpoints affected by the diosgenin in the pathogenesis of breast cancer (*Homo sapiens* (human); *hsa05224*) 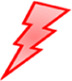
targets/pathways. In the KEGG pathway analysis, it was diosgenin-regulated targets involved in breast cancer were also identified to trigger EGFR, PI3K-Akt, MAPK, and p53 signaling pathways.

During the differential gene expression analysis of diosgenin-regulated genes, we observed that *PDGFRB*, *FBFR2*, and *STAT3* possess maximum gene expression in tumor and metastatic vs*.* normal tissues. In addition, molecular docking and simulation also identified *IGF1R*, *MDM2*, and *SRC* could be the prime diosgenin-modulated targets against breast cancer. Hence, to confirm this we further assessed the functional role of each target using cell line studies. The *PDGF* family (*PDFGRB*) has been reported to use *PDGF* ligands released by cancer stromal cells from breast cancer cells to drive cell proliferation (Farooqi and Siddik, 2015). Similarly, *FBFR2* has been reported to promoting cell self-renewal by interacting with NF-kB signals (Kim et al., 2013). Likewise in previous studies, *STAT3* inhibition has been reported to decrease cell proliferation and apoptosis promotion in various cancers including breast cancer (Kanai et al., 2003; Pancotti et al., 2012; Chen et al., 2013). In this regard, because these targets were directly triggered by diosgenin and were also significantly higher in the tumor than in the normal, diosgenin may limit cell proliferation and promote apoptosis by interfering with the self-renewal process. This hypothesis was further supported by the MTT and scratch assays. Previously, an increase in glucose uptake has been reported in the cancer cell which supports ATP production and acts as a fuel (Adekola et al., 2012). This glucose uptake can be reduced by blocking insulin’s impact on cancer cells. In comparison to metformin, the current investigation found a considerably greater effective concentration to limit glucose uptake. As a result, diosgenin may inhibit glucose uptake in cancer cells. In the current investigation, however, the glucose absorption assay was done in the presence of insulin. As a consequence, more research is needed to determine its role in glucose uptake in the absence of insulin. Similarly, *MDM2* helps cancer cells to escape p53 surveillance and avoid cellular apoptosis (Momand et al., 1998). In the present study, since, diosgenin had a significant binding affinity with *MDM2*, it could probably promote the apoptosis that needs to be further confirmed. In addition, *SRC* has been indicated for cancer metastasis and also helps in cancer progression and development which is an indicator of cell proliferation (Wheeler et al., 2009). In the current investigation, we discovered that diosgenin inhibited cell proliferation in a variety of cell lines. This may be due to the blockage of the *SRC* generated by diosgenin by binding to it. This could have prevented cell multiplication, as revealed by the scratch assay. Based on the information presented above, it is reasonable to believe that diosgenin may act against breast cancer by reducing glucose utilization, invasion, and metastasis *via* the surface protein *IGF1R*. Furthermore, it may limit cancer cell proliferation by acting on another surface protein, *PDGFRB*. In addition, the diosgenin may also act over the *MDM2* and prevent the cancer cell to escape the p53 surveillance in the cytoplasm. Also, diosgenin may primarily act over two cytoplasmic targets *FGFR2* and *SRC* to prevent the interaction with NF-kB (prevents cell self-renewal) and metastatic spread respectively (Figure 14) and may also modulate other pathways within the breast cancer pathogenesis (Figure 15) which was evidenced during KEGG pathway analysis.

Although diosgenin inhibited cell growth, was cytotoxic, and had an effect on the various cellular compartments of the tumor cell, the inhibitory constant efficacy was found to be greater than that of doxorubicin. In addition, the previous drug discovery process utilized the concept of the “*lock and key*” approach in which a designed drug is specific to a single protein e.g. doxorubicin complexes with DNA by intercalation and inhibits topoisomerase II. However, this approach has often failed many times and molecules were potent cytotoxic to normal cells which was also observed in the present study. However, a single compound preferably from a natural source tends to act through a polypharmacology approach in which a single molecule can target multiple proteins based on the concept “*master key can unlock multiple locks*” to target multiple proteins and pathways (Chandran et al., 2017). If it happens, the amount of concentration required is a bit high; however, approaches should be made through the targeted drug delivery to breast cancer which is yet to be studied. This issue could be remedied by increasing diosgenin’s cellular permeability and promoting anti-cancer action against breast tumors *via* a novel drug delivery mechanism, as established in previous studies (Dhamecha et al., 2015; Jagwani et al., 2020; Dhamecha et al., 2021; Ramasamy et al., 2021). In addition, the effect of diosgenin on the hub genes (*SRC*, *MDM2,* and *IGF1R*) expression is based on the computational models which need to be further evaluated using real-time or reverse transcriptase polymerase chain reaction even though the present study pointed their functional effect *via* the glucose uptake, cell proliferation, and apoptosis which is the perspective of the present findings.

## Conclusion

The present study utilized a series of system biology tools to trace the potential action of diosgenin against breast cancer. Herein, we identified the probable action of the diosgenin against breast cancer *via* FoxO, PI3K-Akt, p53, Ras, and MAPK signaling pathways. In addition, we traced the selectivity of the diosgenin to manipulate the action of three hub genes *i.e., IGF1R*, *MDM2*, and *SRC*. Our molecular modeling study reveals that the stable complex formation is primarily facilitated by the cooperative closure motion exerted by the primary binding pocket residues in diosgenin-*MDM2*, and diosgenin-*IGF1R* complex while, in Diosgenin-*SRC* similar compact closure dynamics are also observed at the alternate binding pocket. Conformational flexibility and convergence of the trajectories during MD have been investigated using PCA. Diosgenin showed stable non-bonded interactions forming stable binary complexes with all three screened targets namely *SRC*, *MDM2*, and *IGF1R*. Thus, we proposed that these stable interactions of Diosgenin would trigger the successful inhibition of *SRC*, *MDM2*, and *IGF1R*; the newly identified targets in breast cancer. The results of our computer modeling investigations agree well with the results of the experimental cell line tests. As a result, we hope that this work will open the way for the development of novel therapeutic techniques and/or medication candidates for breast cancer.

## Data Availability

The original contributions presented in the study are included in the article/Supplementary Material, further inquiries can be directed to the corresponding authors.
