# Supplementary material for: Systems and in vitro pharmacology profiling of diosgenin against breast cancer
Source: Front Pharmacol. 2023 Jan 4;13:1052849. doi: 10.3389/fphar.2022.1052849 (PMC9846155; doi:10.3389/fphar.2022.1052849)
Supplement: Supplementary file 1 [file DataSheet1.ZIP › Supplementary data/Supplimentary Documents/Supplimentary Figures.pdf]

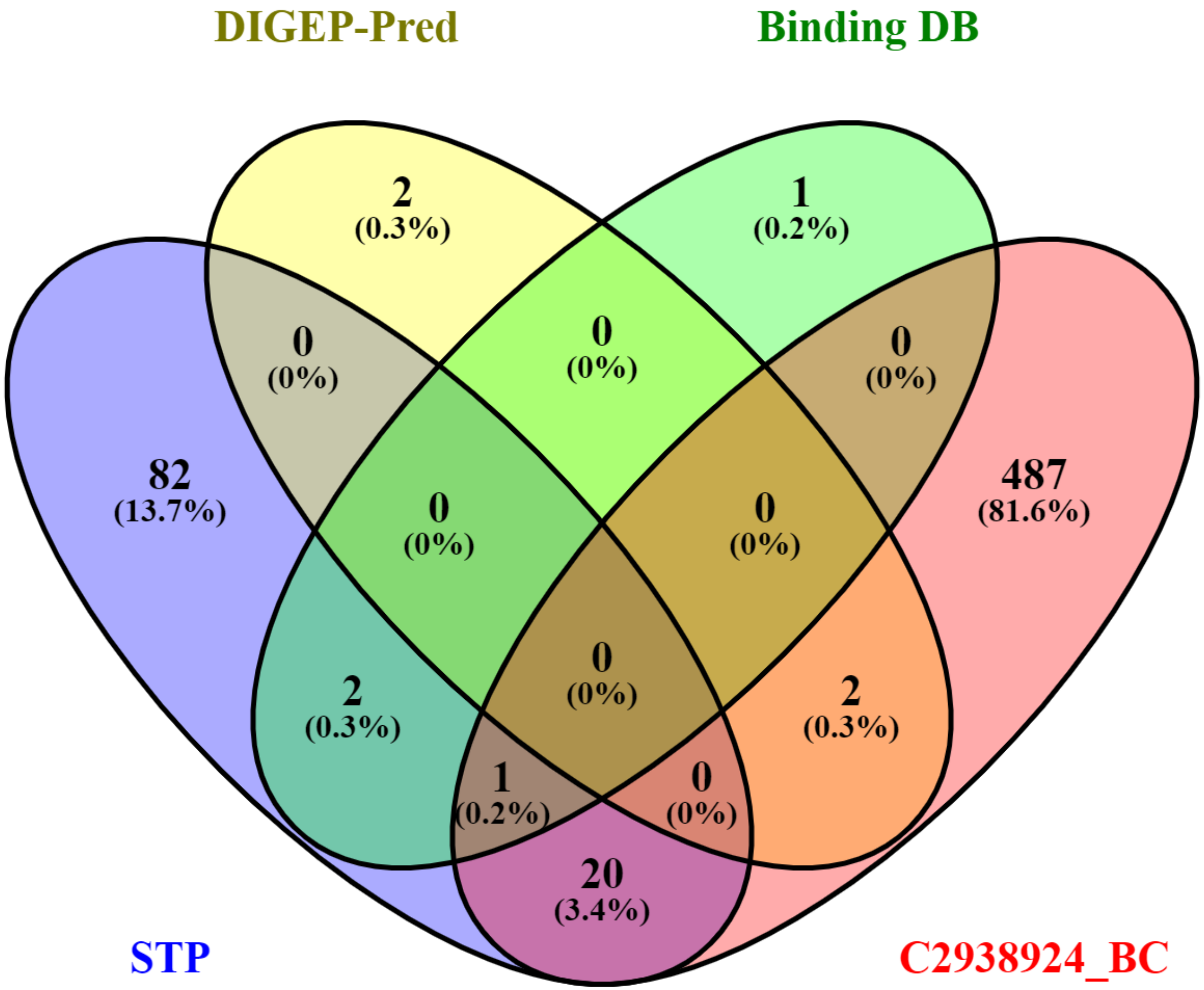

Figure S1: Venn-diagram presenting the diosgenin-modulated common targets from SwissTargetPrediction (STP), DIGEP-Pred, and BindingDB with semantic type (neoplastic process) oestrogen receptor-positive breast cancer (BC) targets (UMLS CUI: ***C2938924)***

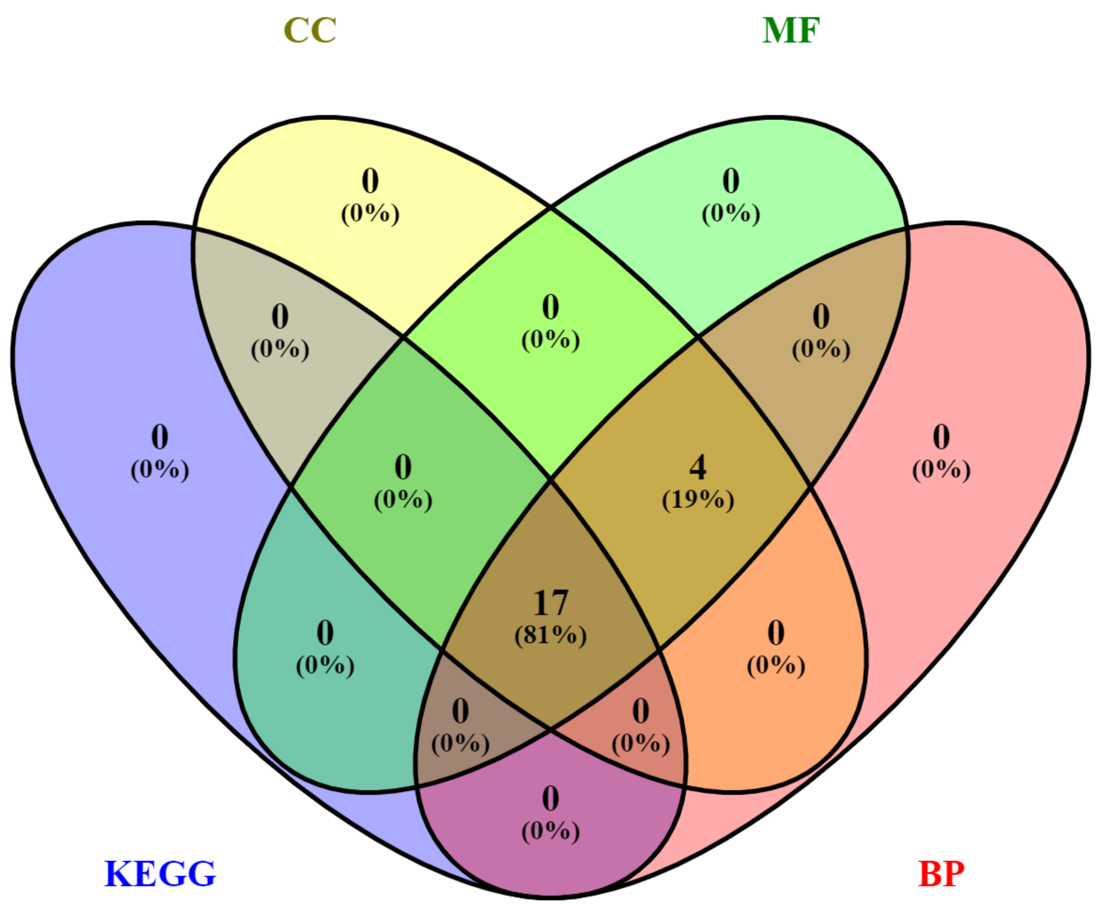

Figure S2: Venn diagram presenting the commonly regulated genes in enrichment analysis; ***CC***: Cellular components, ***MF***: Molecular function, ***KEGG***: Kyoto Encyclopedia of Genes and Genomes, ***BP***: Biological processes

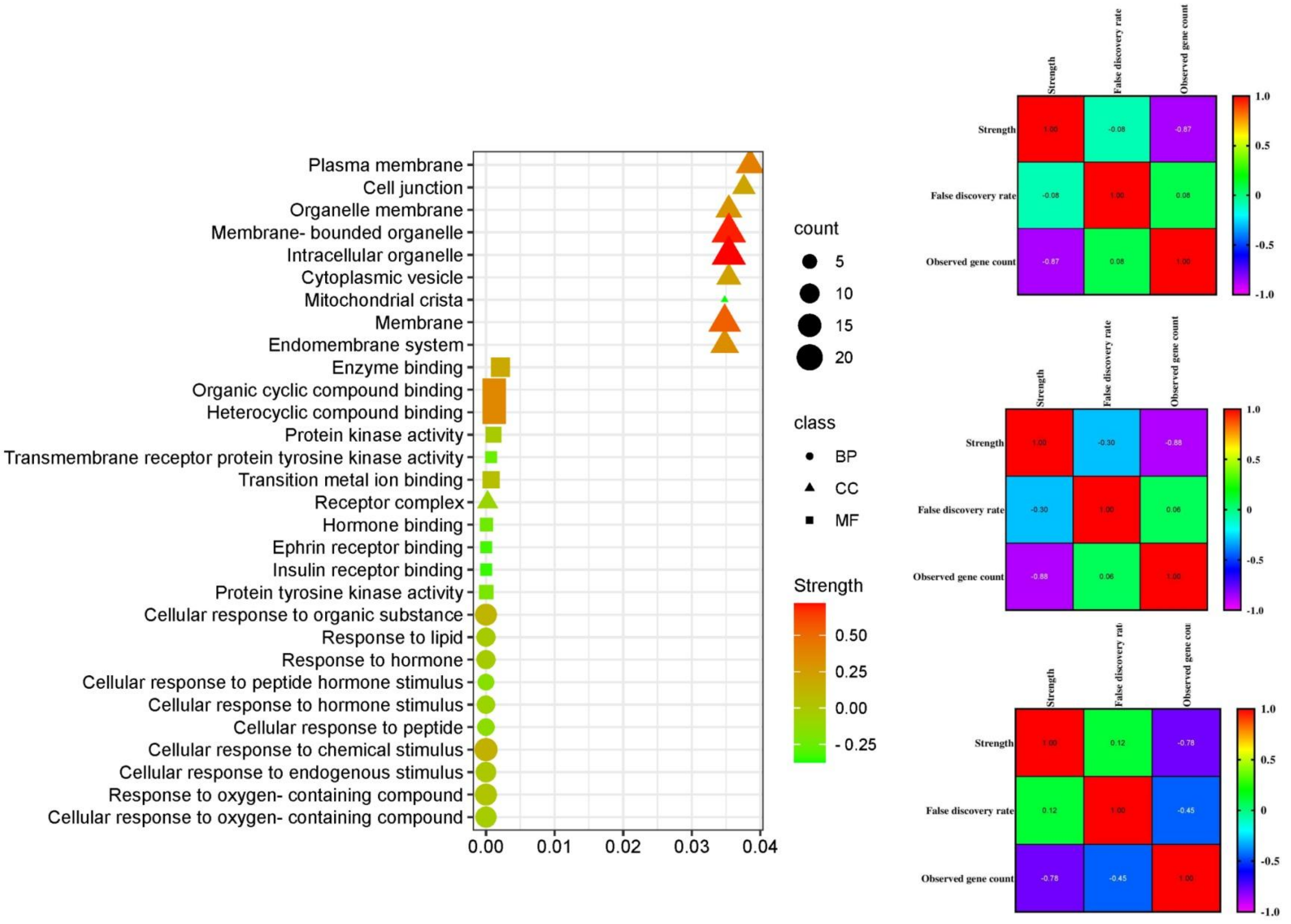

Figure S3: 3 X 3 correlation matrix of (a) cellular components, (b) molecular function, and (c) biological processes of diosgenin-modulated breast cancer targets' gene ontology analysis concerning strength, false discovery rate, and observed gene count.

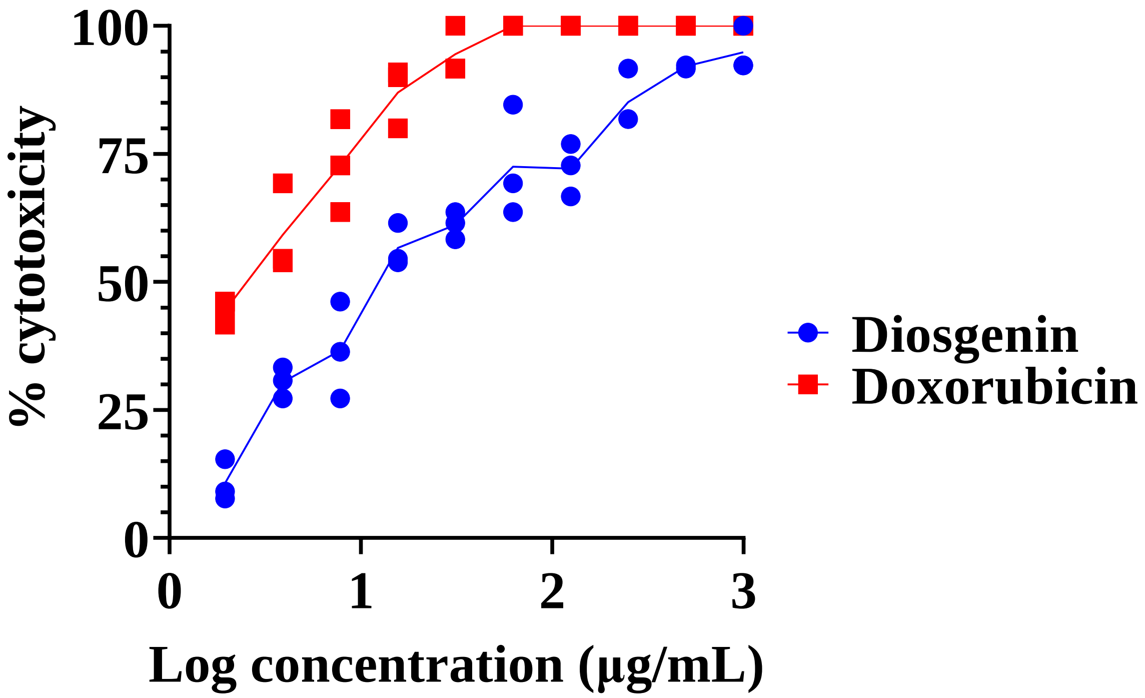

Figure S4: Brine shrimp lethality bioassay of the various concentrations of the diosgenin and doxorubicin in 24 h exposure.

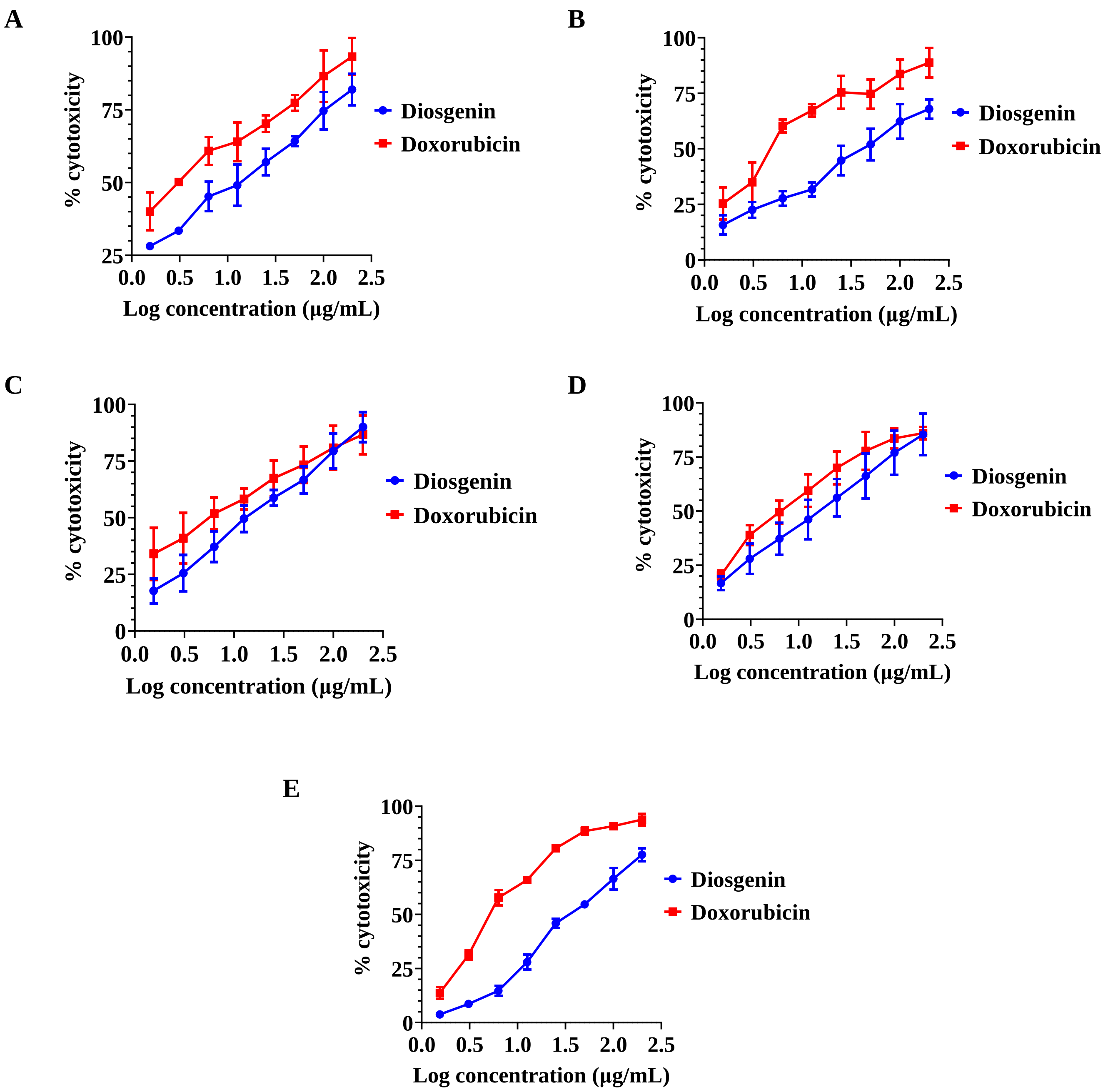

Figure S5: Percentage cytotoxicity *vs* log concentration of diosgenin and doxorubicin over (a) MCF7, **(b)** MDA-MB-231, (c) SKBR3, (d) T47D, and (e) Vero cell lines

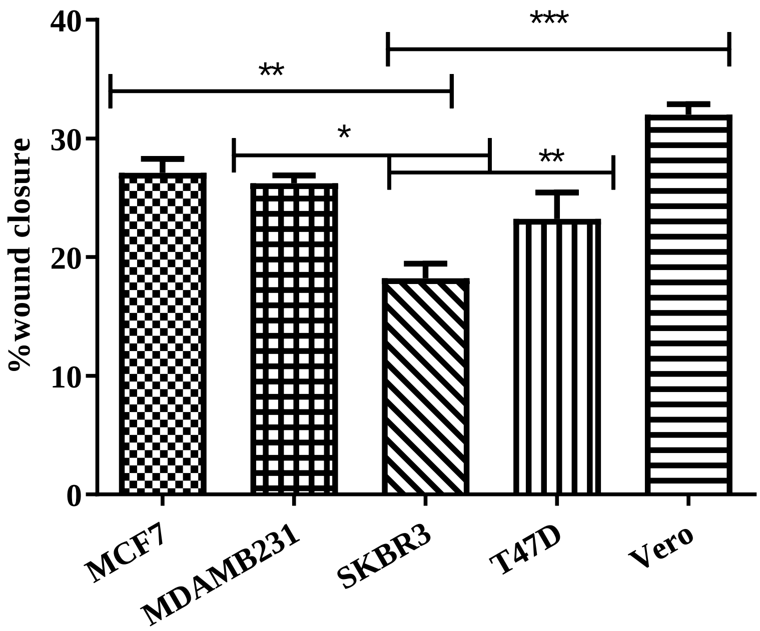

Figure S6: Effect of Diosgenin on scratch closure in different tumor and normal cell lines. Data were analyzed using one-way ANOVA (F= 14.18, R^2= 0.8501) followed by Tukey's multiple comparison post hoc test, *p<0.05, **p<0.01, ***p<0.001.

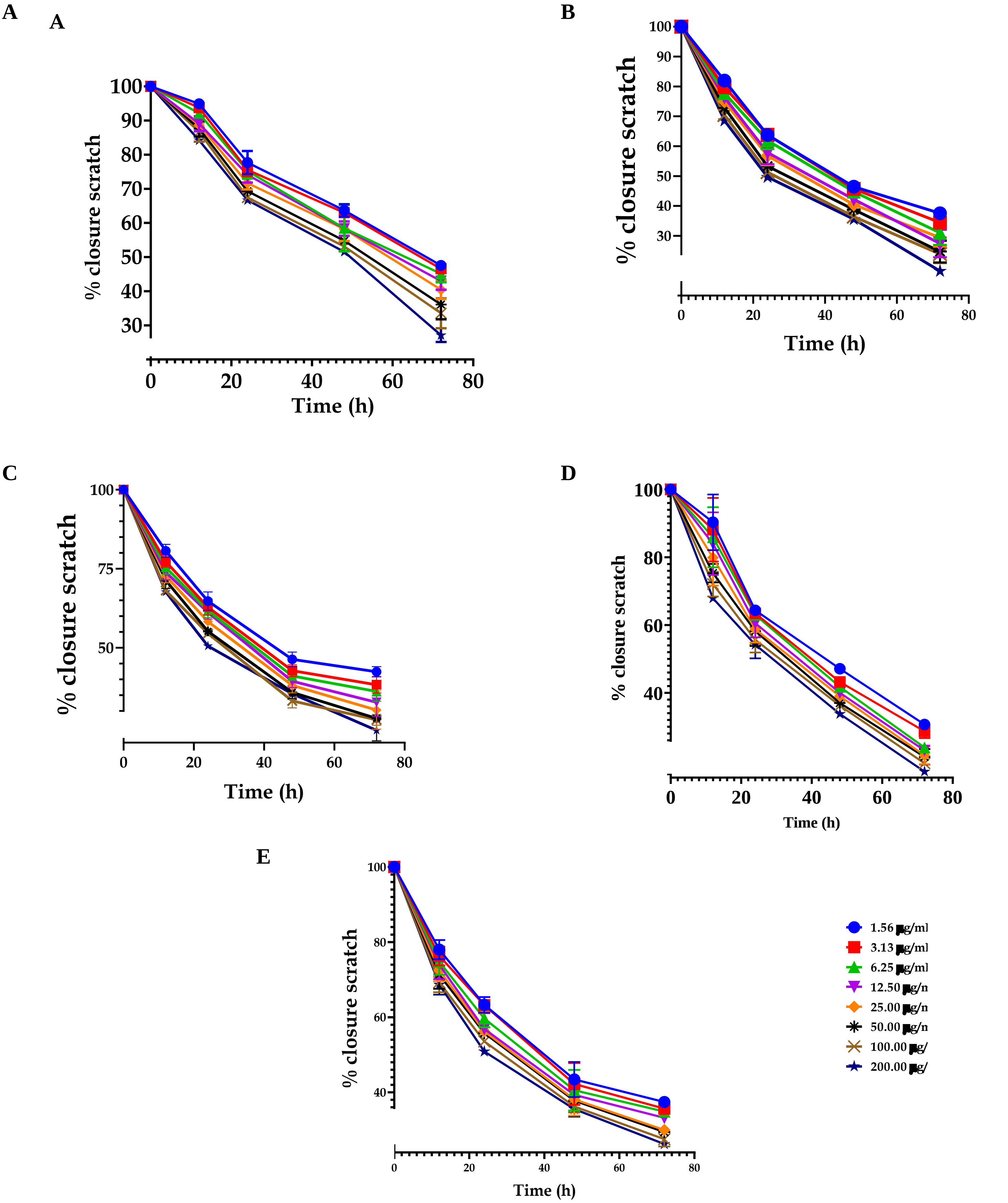

Figure S7: Scratch closure after diosgenin treatment in (a) MCF, (b) SKBR3, (c) T47D, (d) Vero and MDA-MB-231 cell lines

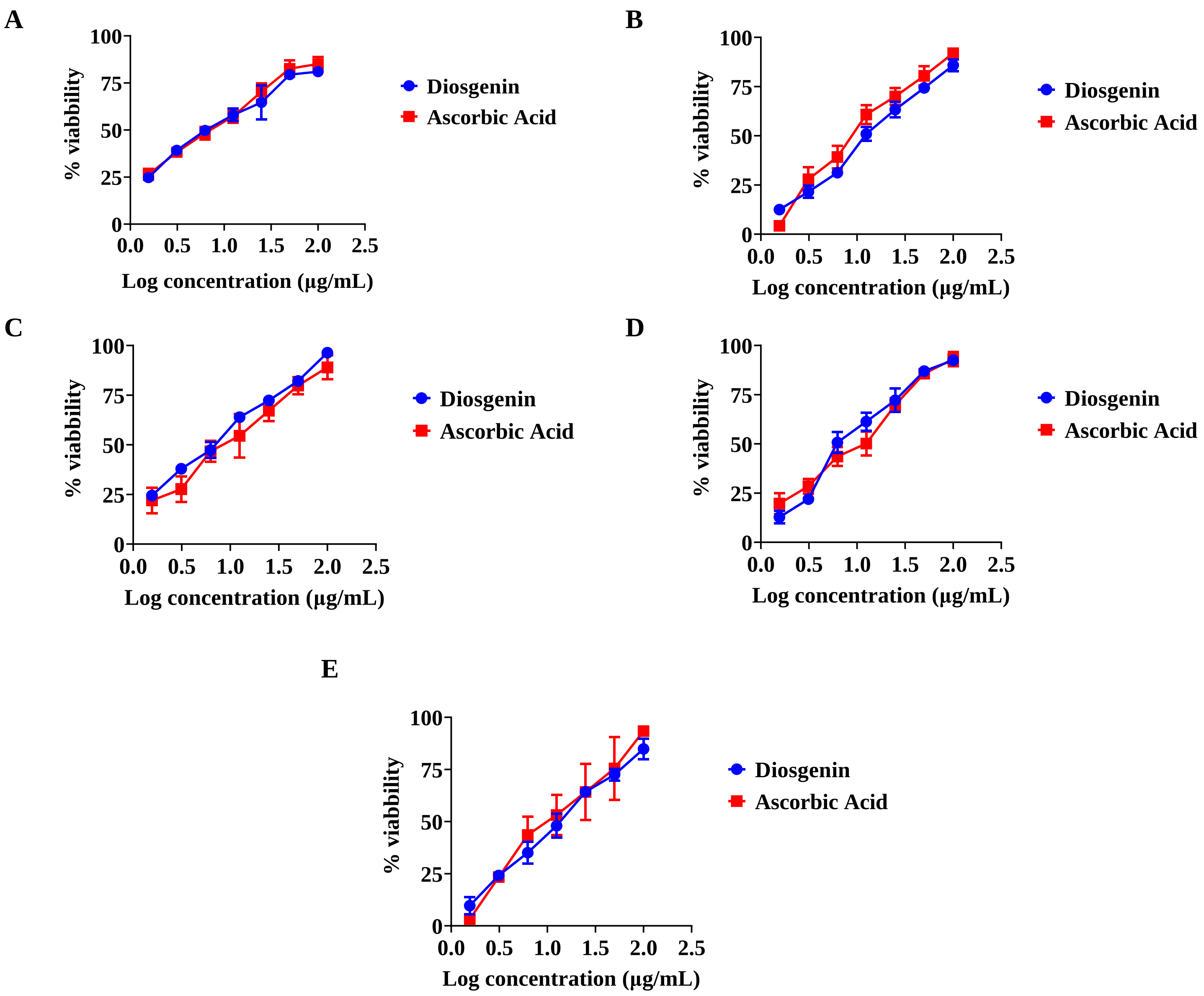

Figure S8: Effect of diosgenin and doxorubicin on % viability in (a) MCF7, (b) MDA-MB-231, (c) SKBR3, (d) T47D, and (e) Vero cell lines

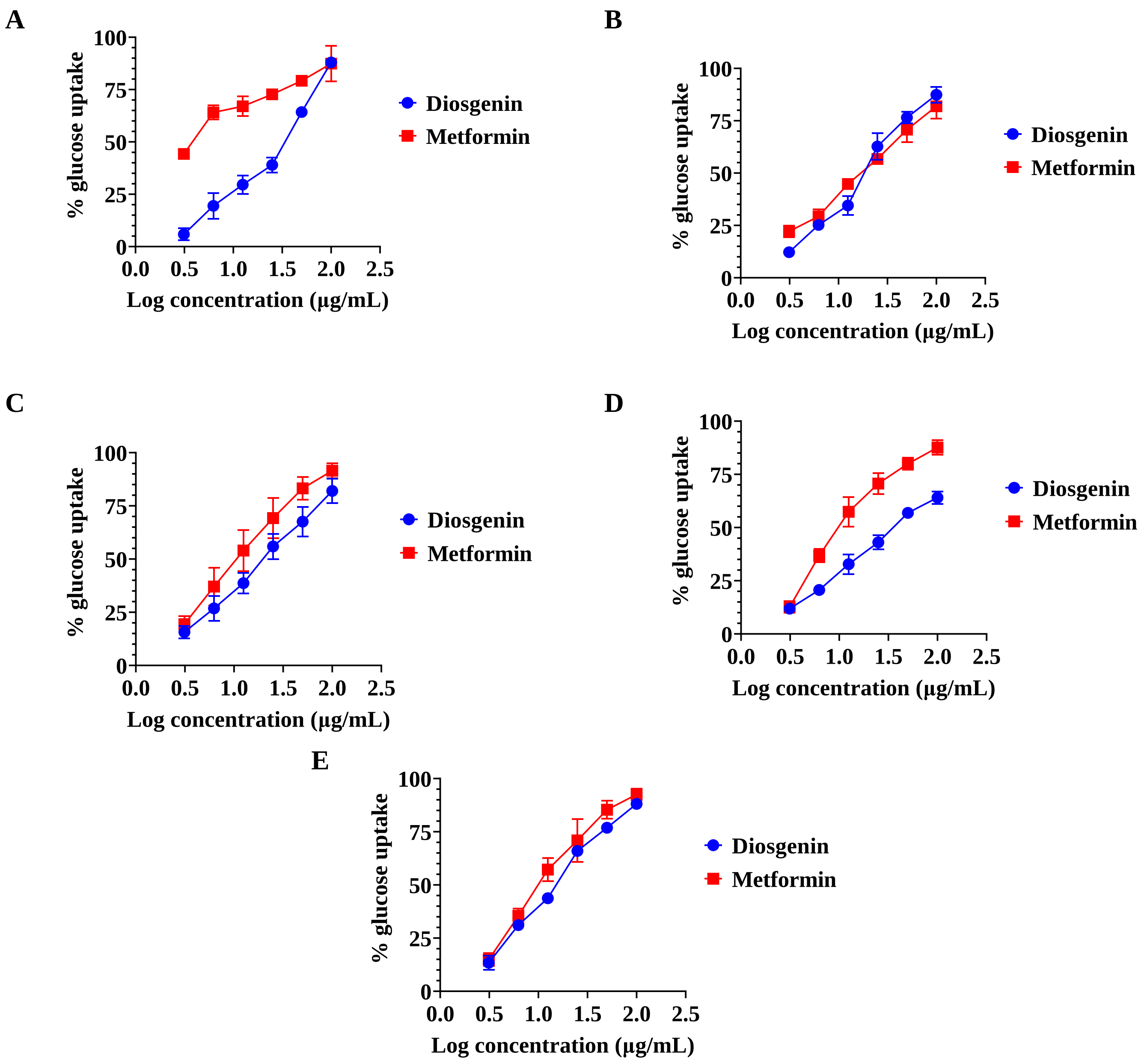

Figure S9: Effect of diosgenin and metformin on the glucose uptake in the presence of insulin in (a) MCF7, (b) MDA-MB-231, (c) SKBR3, (d) T47D, and (e) Vero cell lines
