# Supplementary material for: Systems and in vitro pharmacology profiling of diosgenin against breast cancer
Source: Front Pharmacol. 2023 Jan 4;13:1052849. doi: 10.3389/fphar.2022.1052849 (PMC9846155; doi:10.3389/fphar.2022.1052849)
Supplement: Supplementary file 1 [file DataSheet1.ZIP › Supplementary data/Supplimentary Documents/Supplimentary Tables.docx]

**Table S1: Cluster analysis (K-means) of protein-protein interaction**

| **Clusters** | **Node color** | **Gene count** | **Protein name** |
| --- | --- | --- | --- |
| Cluster 1 | Red | 7 | *AR*, *CYP17A1*, *CYP19A1*, *CYP3A4*, *FASN*, *NR3C1*, *PRCP* |
| Cluster 2 | Green | 12 | *APP*, *CDK4*, *CRHR1*, *FGFR2*, *GRM1*, *IGF1R*, *LYN*, *PDGFRB*, *PTPN1*, *RET*, *SRC*, *STAT3* |
| Cluster 3 | Blue | 2 | *MDM2*, *MDM4* |
| *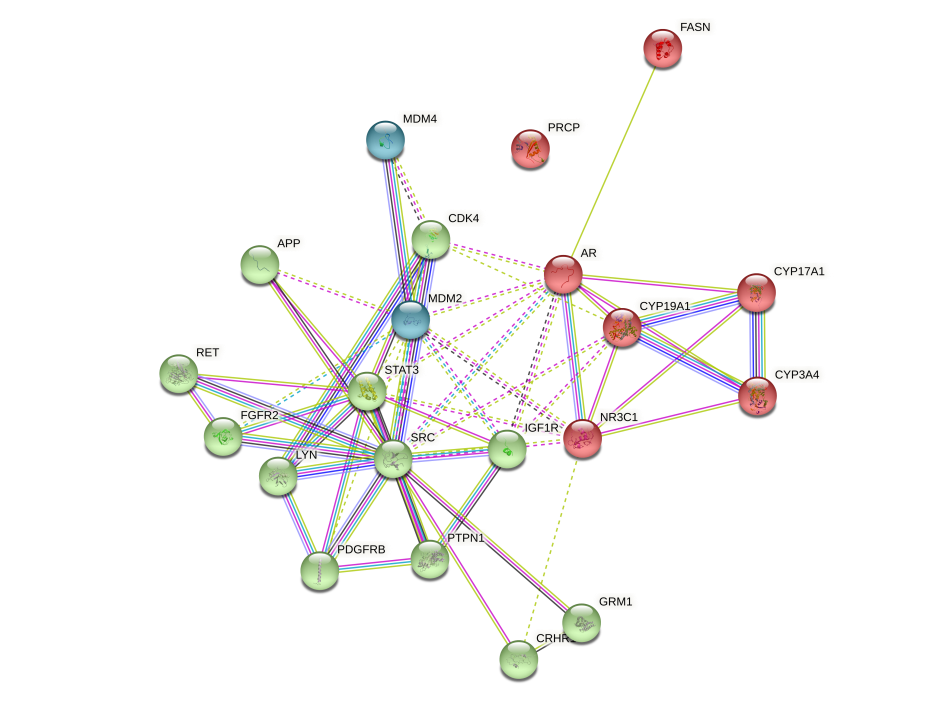*  3 clusters in protein-protein interactions; red (cluster 1), green (cluster 2), and blue (cluster 3). | | | |

**Table S2: Binding free energy and interaction energies of diosgenin with IGF1R, MDM2, and SRC**

| **Complex name** | **MM-PBSA (kcal/mol)** | | | | | |
| --- | --- | --- | --- | --- | --- | --- |
|  | **ΔE_VDW_** | **ΔE_ELE_** | **ΔG_Sol_** | **ΔG_Surf_** | **ΔS** | **ΔG_bind_** |
| IGF1R-Diosgenin | −43.59 ± 4.78 | −4.45 ± 1.28 | 17.63 ± 4.27 | −4.65 ± 0.43 | 0.083 | −35.143±3.03 |
| MDM2-Diosgenin | −40.48 ± 2.73 | −1.67 ± 2.84 | 11.75 ± 3.45 | −4.15 ± 0.17 | 0.069 | −34.619±2.81 |
| SRC-Diosgenin | −25.85 ± 3.05 | −0.73 ± 1.77 | 11.44 ± 6.17 | −2.75 ± 0.27 | 0.094 | −17.994±5.67 |

ΔE_VDW_ = van der Waals contribution; ΔE_ELE_ = electrostatic energy; ΔG_Sol_ = polar solvation free energy; ΔG_Surf_ = solvent-accessible surface area; ΔS=entropy change, ΔG_bind_ = Binding free energy.

**Table S3: Survival data for the diosgenin-regulated targets involved in the breast cancer**

| **Targets** | **Hazard ratio** | **Log-rank P** | **Number at risk** | | | | | | | | | |
| --- | --- | --- | --- | --- | --- | --- | --- | --- | --- | --- | --- | --- |
|  |  |  | **0 month** | | **20 months** | | **40 months** | | **60 months** | | **80 months** | |
|  |  |  | **Low** | **High** | **Low** | **High** | **Low** | **High** | **Low** | **High** | **Low** | **High** |
| APP | 0.69 (0.55-0.87) | 0.0018 | 834 | 2142 | 806 | 2096 | 642 | 1678 | 324 | 820 | 17 | 50 |
| AR | 0.7 (0.56-0.89) | 0.0033 | 848 | 2128 | 819 | 2083 | 601 | 1719 | 266 | 878 | 13 | 54 |
| CDK4 | 1.49 (1.12-1.98) | 0.0062 | 746 | 2230 | 737 | 2165 | 596 | 1724 | 293 | 851 | 16 | 51 |
| CRHR1 | 0.82 (0.65-1.03) | 0.09 | 1762 | 1214 | 1710 | 1192 | 1365 | 955 | 682 | 462 | 39 | 28 |
| CYP17A1 | 0.82 (0.63-1.07) | 0.14 | 2176 | 800 | 2117 | 785 | 1709 | 611 | 852 | 292 | 52 | 15 |
| CYP19A1 | 1.27 (1.01-1.58) | 0.036 | 1614 | 1362 | 1565 | 1337 | 1264 | 1056 | 616 | 528 | 41 | 26 |
| CYP3A4 | 1.29 (0.99-1.67) | 0.056 | 826 | 2150 | 805 | 2097 | 644 | 1676 | 354 | 790 | 30 | 37 |
| FASN | 0.83 (0.65-1.07) | 0.15 | 743 | 2233 | 723 | 2179 | 551 | 1769 | 273 | 871 | 10 | 57 |
| FGFR2 | 0.61 (0.49-0.77) | 2.90E-05 | 802 | 2174 | 771 | 2131 | 598 | 1722 | 305 | 839 | 18 | 49 |
| GRM1 | 1.28 (1.03-1.61) | 0.027 | 1768 | 1208 | 1732 | 1170 | 1370 | 950 | 667 | 477 | 34 | 33 |
| IGF1R | 0.62 (0.5-0.78) | 3.20E-05 | 922 | 2054 | 891 | 2011 | 726 | 1594 | 354 | 790 | 21 | 46 |
| LYN | 1.33 (1.05-1.68) | 0.017 | 2121 | 855 | 2075 | 827 | 1663 | 657 | 830 | 314 | 46 | 21 |
| MDM2 | 0.69 (0.55-0.87) | 1.20E-03 | 929 | 2047 | 894 | 2008 | 746 | 1574 | 406 | 738 | 31 | 36 |
| MDM4 | 0.8 (0.64-0.99) | 0.044 | 1311 | 1665 | 1272 | 1630 | 1090 | 1230 | 585 | 559 | 46 | 21 |
| NR3C1 | 0.66 (0.52-0.83) | 3.80E-04 | 1623 | 1353 | 1576 | 1326 | 1262 | 1058 | 614 | 530 | 33 | 34 |
| PDGFRB | 0.58 (0.46-0.72) | 1.20E-06 | 1271 | 1705 | 1225 | 1677 | 982 | 1338 | 470 | 674 | 29 | 38 |
| PRCP | 0.74 (0.58-0.94) | 1.50E-02 | 1930 | 1046 | 1879 | 1023 | 1510 | 810 | 727 | 417 | 35 | 32 |
| PTPN1 | 1.53 (1.21-1.94) | 4.10E-04 | 2213 | 763 | 2161 | 741 | 1765 | 555 | 907 | 237 | 52 | 15 |
| RET | 1.28 (1.02-1.6) | 3.0E-02 | 1720 | 1256 | 1682 | 1220 | 1328 | 992 | 654 | 490 | 39 | 28 |
| SRC | 0.71 (0.57-0.9) | 3.90E-03 | 897 | 2079 | 865 | 2037 | 727 | 1593 | 354 | 790 | 17 | 50 |
| STAT3 | 0.57 (0.46-0.72) | 1.40E-06 | 806 | 2170 | 773 | 2129 | 605 | 1715 | 289 | 855 | 17 | 50 |

**Table S4: IC_50_ of the diosgenin and doxorubicin over the various cell lines identified through the MTT assay**

| Cell lines | Inhibitory constant (IC_50_) in µg/mL | |
| --- | --- | --- |
|  | Diosgenin | Doxorubicin |
| MCF7 | 12.05±1.33*** | 3.21±0.29 |
| MDA-MB-231 | 45.54±23.41* | 6.30±2.67 |
| SKBR3 | 15.11±5.32* | 5.47±1.09 |
| T47D | 17.78±7.86 | 8.18±2.36 |
| Vero | 38.59±4.03*** | 7.05±0.69 |

*p<0.05, **p<0.01, ***p<0.001 compared to IC_50_ of doxorubicin on respective cell line

**Table S5: IC_50_ of diosgenin *vs* ascorbic acid over H_2_O_2_-induced stress in cell lines**

| Cell lines | Inhibitory constant (IC_50_) in µg/mL | |
| --- | --- | --- |
|  | Diosgenin | Ascorbic acid |
| MCF7 | 7.68±0.51 | 7.13±0.31 |
| MDA-MB-231 | 13.58±0.90* | 10.60±1.60 |
| SKBR3 | 6.68±0.67 | 9.39±3.09 |
| T47D | 8.90±0.98 | 9.14±0.78 |
| Vero | 13.72±1.83 | 12.68±4.53 |

*p<0.05 compared to IC50 of ascorbic acid in MDA-MB-231 cell lines

**Table S6: Effect of diosgenin and metformin against Warburg effect**

| Cell lines | Inhibitory constant (EC_50_) in µg/mL | |
| --- | --- | --- |
|  | Diosgenin | Metformin |
| MCF7 | 26.19±2.77*** | 3.10±0.99 |
| MDA-MB-231 | 17.92±1.19 | 17.03±2.59 |
| SKBR3 | 19.99±2.91* | 11.74±3.19 |
| T47D | 37.47±1.75*** | 12.50±1.42 |
| Vero | 15.27±0.95** | 11.79±1.65 |

*p<0.05, **p<0.01, ***p<0.001 compared to EC50 of metformin on respective cell line
